# Discovery and replication of SNP-SNP interactions for quantitative lipid traits in over 60,000 individuals

**DOI:** 10.1186/s13040-017-0145-5

**Published:** 2017-07-24

**Authors:** Emily R. Holzinger, Shefali S. Verma, Carrie B. Moore, Molly Hall, Rishika De, Diane Gilbert-Diamond, Matthew B. Lanktree, Nathan Pankratz, Antoinette Amuzu, Amber Burt, Caroline Dale, Scott Dudek, Clement E. Furlong, Tom R. Gaunt, Daniel Seung Kim, Helene Riess, Suthesh Sivapalaratnam, Vinicius Tragante, Erik P.A. van Iperen, Ariel Brautbar, David S. Carrell, David R. Crosslin, Gail P. Jarvik, Helena Kuivaniemi, Iftikhar J. Kullo, Eric B. Larson, Laura J. Rasmussen-Torvik, Gerard Tromp, Jens Baumert, Karen J. Cruickshanks, Martin Farrall, Aroon D. Hingorani, G. K. Hovingh, Marcus E. Kleber, Barbara E. Klein, Ronald Klein, Wolfgang Koenig, Leslie A. Lange, Winfried Mӓrz, Kari E. North, N. Charlotte Onland-Moret, Alex P. Reiner, Philippa J. Talmud, Yvonne T. van der Schouw, James G. Wilson, Mika Kivimaki, Meena Kumari, Jason H. Moore, Fotios Drenos, Folkert W. Asselbergs, Brendan J. Keating, Marylyn D. Ritchie

**Affiliations:** 1 Computational and Statistical Genomics Branch, National Human Genome Research Institute, National Institute for General Medical Sciences, National Institutes of Health, Baltimore, MD USA; 2 The Center for Systems Genomics, The Pennsylvania State University, University Park, State College, PA USA; 3 Department of Surgery, Duke University, Durham, NC USA; 4 Department of Genetics, Geisel School of Medicine at Dartmouth, Hanover, NH USA; 5 Department of Epidemiology, Geisel School of Medicine at Dartmouth, Hanover, NH USA; 6 Department of Medicine, McMaster University, Hamilton, ON Canada; 7 Department of Lab Medicine and Pathology, University of Minnesota, Minneapolis, MN USA; 8 London School of Hygiene and Tropical Medicine, London, UK; 9 Division of Medical Genetics, Department of Medicine, University of Washington, Seattle, WA USA; 10 MRC Integrative Epidemiology Unit, University of Bristol, Oakfield House, Oakfield Grove, Bristol, UK; 11 Institute of Epidemiology II, Helmholtz Zentrum München, German Research Center for Environmental Health, Neuherberg, Germany; 12 Department of Vascular Medicine, Academic Medical Center, Amsterdam, The Netherlands; 13 Department of Cardiology, Division Heart and Lungs, University Medical Center Utrecht, Utrecht, The Netherlands; 14 Department of Medical Genetics, Biomedical Genetics, University Medical Center Utrecht, Utrecht, The Netherlands; 15 Durrer Center for Cardiogenetic Research, ICIN-Netherlands Heart Institute, Utrecht, The Netherlands; 16 Department of Clinical Epidemiology, Biostatistics and Bioinformatics, Academic Medical Center, Amsterdam, The Netherlands; 17 Department of Medical Genetics, Marshfield Clinic, Marshfield, WI USA; 18 Group Health Research Institute, Group Health Cooperative, Seattle, WA USA; 19 Division of Molecular Biology and Human Genetics, Department of Biomedical Sciences, Stellenbosch University, Tygerberg, South Africa; 20 Division of Cardiovascular Diseases, Mayo Clinic, Rochester, MN USA; 21 Department of Preventive Medicine, Northwestern University Feinberg School of Medicine, Chicago, IL USA; 22 Department of Population Health Sciences, Department of Ophthalmology and Visual Sciences, University of Wisconsin-Madison, Madison, WI USA; 23 Department of Cardiovascular Medicine, The Wellcome Trust Centre for Human Genetics, University of Oxford, Oxford, UK; 24 Department of Epidemiology and Public Health, UCL Institute of Epidemiology & Health Care, University College London, London, UK; 25 Vth Department of Medicine, Medical Faculty Mannheim, Heidelberg University, Heidelberg, Germany; 26 Department of Internal Medicine II – Cardiology, University of Ulm Medical Centre, Ulm, Germany; 27 Department of Genetics, University of North Carolina School of Medicine at Chapel Hill, Chapel Hill, NC USA; 28 Synlab Academy, Synlab Services GmbH, Mannheim, Germany; 29 Department of Epidemiology, School of Public Health, University of North Carolina at Chapel Hill, Chapel Hill, NC USA; 30 Julius Center for Health Sciences and Primary Care, University Medical Center Utrecht, Utrecht, The Netherlands; 31 Division of Public Health Sciences, Fred Hutchinson Cancer Research Center, Seattle, WA USA; 32 MRC Integrative Epidemiology Unit, School of Social and Community Medicine, University of Bristol, Bristol, UK; 33 Department of Physiology and Biophysics, University of Mississippi Medical Center, Jackson, MS USA; 34 ISER, University of Essex, Essex, UK; 35 Institute for Biomedical Informatics, Perelman School of Medicine, University of Pennsylvania, Philadelphia, PA USA; 36 Centre of Cardiovascular Genetics, Institute of Cardiovascular Science, Faculty of Population Health Sciences, University College London, London, UK; 37 Division of Genetics, The Children’s Hospital of Philadelphia, Philadelphia, PA USA; 38 Division of Transplantation, Department of Surgery, University of Pennsylvania, Philadelphia, PA USA; 39 Biomedical and Translational Informatics, Geisinger Clinic, Danville, PA USA

**Keywords:** Genetics, Lipids, Interactions, Computational genetics, Genetic epidemiology

## Abstract

**Background:**

The genetic etiology of human lipid quantitative traits is not fully elucidated, and interactions between variants may play a role. We performed a gene-centric interaction study for four different lipid traits: low-density lipoprotein cholesterol (LDL-C), high-density lipoprotein cholesterol (HDL-C), total cholesterol (TC), and triglycerides (TG).

**Results:**

Our analysis consisted of a discovery phase using a merged dataset of five different cohorts (*n* = 12,853 to *n* = 16,849 depending on lipid phenotype) and a replication phase with ten independent cohorts totaling up to 36,938 additional samples. Filters are often applied before interaction testing to correct for the burden of testing all pairwise interactions. We used two different filters: 1. A filter that tested only single nucleotide polymorphisms (SNPs) with a main effect of *p* < 0.001 in a previous association study. 2. A filter that only tested interactions identified by Biofilter 2.0. Pairwise models that reached an interaction significance level of *p* < 0.001 in the discovery dataset were tested for replication. We identified thirteen SNP-SNP models that were significant in more than one replication cohort after accounting for multiple testing.

**Conclusions:**

These results may reveal novel insights into the genetic etiology of lipid levels. Furthermore, we developed a pipeline to perform a computationally efficient interaction analysis with multi-cohort replication.

**Electronic supplementary material:**

The online version of this article (doi:10.1186/s13040-017-0145-5) contains supplementary material, which is available to authorized users.

## Background

For this study, we perform several analyses to identify and validate genetic interactions associated with circulating lipid levels. Our motivation for studying the contribution of interactions to lipid levels is three-fold. First, dyslipidemia have a large impact on human health. Circulating lipid levels, such as high-density lipoprotein cholesterol (HDL-C), low-density lipoprotein cholesterol (LDL-C), total cholesterol (TC), and triglycerides (TG), are associated with risk for various common disease traits including cardiovascular disease [1]. Cardiovascular disease is the leading cause of death for individuals in developed countries [2]. Secondly, the estimated genetic component for lipid levels is relatively large and highly variable. While age, sex, body mass index (BMI), diet, exercise and smoking status have been shown to have an effect on lipid levels, it is estimated that genetic factors contribute between 40 and 60% overall to variation in lipid levels [3, 4]. A more thorough understanding of the genetics underlying individual variation in lipid levels will result in greater insight into the biological processes underpinning dyslipidemia, and may inform more effective therapies to ultimately lower risk for cardiovascular disease. Lastly, a large portion of the estimated genetic component has not been identified by main effects alone. For the past decade or so, large efforts have been undertaken to tease apart the genetic etiology of common, complex traits, such as circulating lipid level and CVD; however a large proportion of the estimated heritability of these traits that remains unexplained [5, 6]. Sources of missing heritability are likely to be caused by rare variants, epigenetics, structural variation, gene-gene interactions, gene-environment interactions, and/or the accuracy of the heritability models [7, 8]. Notably, calculating the total heritability and measuring the exact contribution of these specific findings to heritability remains a controversial and complex issue [9–12]. However, the consistently small proportion explained by common variants identified by GWAS across all complex traits suggests that we still have a lot to learn about the genetic architecture of these traits. This study addresses the contribution of interactions to the genetic architecture of lipid traits by examining SNP-SNP interactions in four quantitative lipid traits – HDL-C, LDL-C, TC, and TG.

Here we are trying to identify genetic interactions by searching for statistical interactions. For interpretation purposes, it is important to understand how we define an interaction. Biologically, we are trying to identify genetic variants that alter the phenotype in a manner that is dependent on genotypes at two different loci. For example, an individual may have variants in two different regions of a metabolic enzyme protein that cause triglyceride levels to increase even more than the combined additive effects of the variants. Statistically, we use a likelihood ratio test to assess the significance of including a multiplicative interaction term along with the two main effect terms in a linear regression model. While there has been some debate about the relationship between statistical and biological interactions, there is substantial evidence that this method is robust to the non-linear or interaction effects we are interested in [13].One of the main considerations for a genome-wide interaction study (GWIS) is the computational and statistical burden of exhaustive interaction testing, which inherently results in a massive increase in the number of tests (e.g. 1000 SNPs = 499,500 two-way interactions and 166,167,000 three-way interactions, etc.). While our analysis is not a GWIS per se*,* as most individuals were genotyped using a cardiovascular gene-centric array and we filtered before interaction testing, the considerations about interaction do still apply [14]. One approach to address this issue is to filter on main effect significance (i.e. the *p*-value from the main effect term in a regression model) using bona fide index lipid signals derived from existing GWAS. A limitation to the main effect filter approach is that SNPs involved in true interactions with little or no main effects will likely be filtered out. Another approach is to select SNP-SNP models based on knowledge-driven biologically plausible genes/loci, such as selecting SNP pairs in genes shown to physically interact in previous biological experiments (Biofilter) [15].

For this analysis, we used both of the aforementioned filter methods to test for interactions. After applying these filters, we identified potential SNP-SNP interactions for each of the lipid traits in a discovery analysis, which consisted of five cohorts merged into one dataset (*n* = 12,853 to *n* = 16,849 depending on lipid phenotype): Atherosclerosis Risk In Communities (ARIC); Coronary Artery Risk Development in Young Adults (CARDIA); Cardiovascular Health Study (CHS); Framingham Heart Study (FHS); and Multi-Ethnic Study of Atherosclerosis (MESA); from the NHLBI Candidate gene Association Resource (CARe). Models were selected for replication testing based on statistical significance from the discovery set. There were ten replication sets in total with sample sizes between *n* = 1568 and *n* = 7504 totaling 36,938 for the replication dataset. We identified models with the most evidence for significant associations with the lipid traits according multiple-testing corrected likelihood ratio test *p*-value thresholds from linear regression models. We also assessed the number of cohorts in which the models replicated. This study highlights an analysis strategy to explore genetic interactions for complex traits and suggests several replicating interactions for lipid traits.

## Methods

### Discovery: Cohort descriptions

The discovery dataset for each of the traits had n ~ 14,000, with each of the cohorts having the following contributions: ARIC (*n* = 11,906), CARDIA (*n* = 2319), CHS (*n* = 4490), FHS (*n* = 1467), and MESA (*n* = 5598). Individuals were genotyped using the gene-centric ITMAT-Broad-CARe (IBC) array [16], which was previously used in a meta-analysis of 32 studies (66,240 individuals), identifying and replicating many known and novel lipid signals [17]. All of the individuals in our analysis were self-reported European ancestry, subsequently verified using principal component analyses by selecting individuals that clustered with the CEU panel from HapMap, and ≥21 years of age. HDL-C, LDL-C, TC, and TG levels were measured from baseline or first measurement blood samples. All lipid measurements were converted to mmol/L. LDL-C was calculated according to Friedewald’s formula *L ~ C – H - kT* where *C* is total cholesterol, *H* is HDL, *L* is LDL, *T* are triglycerides, and k is 0.45 for mmol/L (or 0.20 if measured in mg/dl). If TG values were >4.51 mmol/L (>400 mg/dL), then LDL was treated as a missing value. More details for the five merged discovery cohorts are shown in Table 1.

**Table 1:** Details for the five cohorts that were merged to create the discovery dataset and the 10 cohorts used for replication

	Study (One letter label)	Recruitment design	Year of collection	N total^a^	Data Level	Study Ref (PMID)
Discovery (IBC)	ARIC	Community-based	1985–2006	9588	Individual	20400780
	CARDIA	Community-based	1985–2003	1443	Individual	20400780
	CHS	Community-based	1988–2005	3952	Individual	20400780
	FHS	Community-based	1948-present	7556	Individual	20,400,780
	MESA	Community-based	1999–2009	2298	Individual	20400780
Replication (IBC)	BOSS/EHLS/BDES	Population-based cohort	1988-present	1568	Summary	21339392, 9801018, 1923372
	BWHHS (B)	Population-based cohort	1999–2001	3411	Summary	16045529
	CLEAR	Case-control	2005	1591	Summary	16474172
	EPIC-NL	Nested case-control	1993–1997	5194	Summary	19483199
	GIRaFH	Cohort	1999	1694	Summary	15554949
	KORA	Population-based cohort	1984–2005	1849	Summary	16032513, 1603251
	LURIC (L)	Case-control	1997–2002	2813	Summary	11258203
	PROCARDIS (P)	Case-control	1998-present	6432	Summary	20032323
	Whitehall II (W)	Population-based cohort	1985–1989	4882	Summary	15576467
Rep. (GWAS)	eMERGE	Consortium		7504	Summary	23743551

Discovery cohorts: Atherosclerosis Risk In Communities (ARIC); Coronary Artery Risk Development in Young Adults (CARDIA); Cardiovascular Health Study (CHS); Framingham Heart Study (FHS); Multi-Ethnic Study of Atherosclerosis (MESA)

Replication cohorts: *BOSS* beaver dam offspring study, *EHLS* epidemiology of hearing loss study, *BDES* beaver dam eye study, *AIBIII* Allied Irish Bank Workers Study III, *AMC-PAS* Academic Medical Center Amsterdam Premature Atherosclerosis Study, *ASCOT* anglo-scandinavian cardiac outcomes trial, *BHS* bogalusa heart study, *BRIGHT*, British genetics of hypertension, *BWHHS* British women’s heart and health study, *CLEAR* carotid lesion epidemiology and risk, *EPIC-NL* European Prospective Investigation into Cancer and Nutrition in the Netherlands, *GIRaFH* genetic identification of risk factors in familial hypercholesterolemia, *KORA* Kooperative Gesundheitsforschung in der Region Augsburg, *LURIC* Ludwigshafen Risk and Cardiovascular Health Study, *PROCARDIS* precocious coronary artery disease study, *WHII* Whitehall II study, *GWAS* eMERGE

^a^Numbers varied for each lipid trait. The number shown is the maximum number of non-missing individuals for all traits

### Discovery: Quality control and statistical analyses

**Fig. 1:**
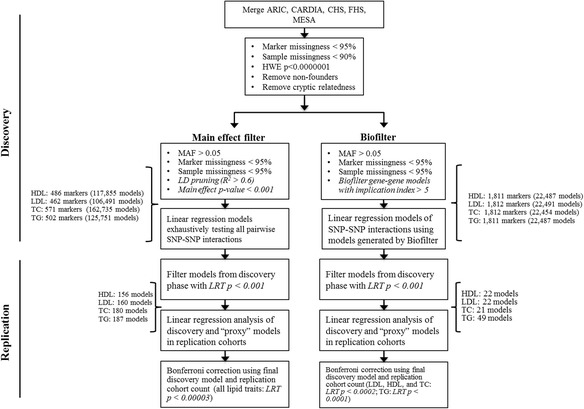
Flowchart of the quality control and analysis steps for the discovery and replication phases

Individuals were genotyped on the ITMAT-Broad-CARe (IBC) array. This array consists of ~50,000 SNPs across ~2100 loci. Selection criteria for SNPs to be included on the IBC array have been described in detail previously [16]. Quality control filters were applied after the cohorts were merged into the full discovery dataset. A summary of the full quality control and analysis pipeline is shown in Fig. 1. All quality control procedures were implemented with the PLINK software package [18] unless otherwise specified. SNPs with a genotype missing rate > 95% or that were not in Hardy-Weinberg equilibrium (*p* < 1.0 x < 10^−7^) were removed from the analysis. After SNP genotyping quality control, 44,750 markers remained. Individuals with SNP genotype missing rates >90% were excluded from the analysis. For cohorts that contained known trios, non-founders (i.e. offspring) were removed. To address unknown or cryptic relatedness, identity-by-descent (IBD) estimates were calculated, and one individual from each pair with pi-hat >0.3 was removed. The TG values were log transformed to improve normality. Four new datasets were created for each of the quantitative lipid traits: HDL-C (*n* = 13,030), LDL-C (*n* = 12,853), TC (*n* = 16,849), and TG (*n* = 13,031).

Additional quality control metrics were applied to the individual lipid datasets for each of the statistical analyses. For both the main effect filter and Biofilter analyses, SNPs with missing phenotypes were removed along with variants with minor allele frequency (MAF) < 0.05 or missing genotype rate > 5%. For the main effect filter analysis, SNPs were pruned to remove high levels of SNP correlation, or LD from the data. No LD pruning was done for the Biofilter interaction analyses, as these models are specifically generated using SNPs that are in different genes. This was performed by removing one SNP from all pairs of SNPs with an r^2^ > 0.6 using PLINK. SNPs with a main effect *p* < 0.001 based on a previous GWAS regression analysis were selected for interaction testing [17]. We had two specific motivations for selecting this threshold for our study: 1. to allow for interactions that may be present in the absence of large, genome-wide significant main effects, and 2. to reduce the SNP set to a size that allowed for a manageable exhaustive SNP-SNP interaction analysis. SNP-SNP models were generated by creating an exhaustive list of all SNP pairs. Importantly, we did not LD prune for the Biofilter analysis due to the method used to generate SNP-SNP models. Biofilter 2.0 is a software package that identifies SNP-SNP models based on probable gene-gene interactions identified in various online sources including Gene Ontology GO and KEGG. The Biofilter method has previously been described in greater detail [15, 19]. Briefly, SNPs are mapped to genes using a 50 kb upstream or downstream inclusion criterion. Gene pairs that may be more likely to interact are then identified in various curated biological knowledge databases. A score is given based on the number of sources that indicate a possible interaction. For this analysis, models were included if at least five knowledge sources identified the gene-gene interaction model. The SNPs are then mapped back to the genes to create the SNP-SNP models for statistical testing.

To test for SNP-SNP interactions, we used an R script that automatically tests the models according to user input parameters [20]. We tested for significant interactions using a linear regression framework. We adjusted for age, sex, smoking status, type 2 diabetes status, BMI, medication use (use or no use of lipid lowering drugs), and potential population substructure (top 10 principal components) by including these as covariate terms in the linear regression models for each of the four lipid traits. We included these covariates to control for any factors outside of genetics that may have an effect on lipid levels and to remain consistent with the previous GWAS from which the SNPs for the main effect filter analysis were chosen. In the previous study that used the same lipid measurements for a gene-centric meta-analysis of main effects [17], an additional adjustment for medication was done by multiplying a constant percentage to account for lipid lowering medication. The two adjustment methods (covariate and multiplication) gave similar results; therefore, we only included the covariate adjustment results in this manuscript. We chose to include the top 10 principal components to remain consistent with the previous GWAS and to control for any residual variation as we were performing these analyses in a combined cohort that included individuals from various parts of the country. Models were selected for replication testing with likelihood ratio test *p* -values <0.001 (comparing the full and reduced linear regression models (Eqs. 1 and 2)). We adjusted the threshold using a Bonferroni correction based on the total number of number of models that were tested for each filtering methods. We estimated these models to be independent due to the LD-pruning in the main effect filter analysis and the SNPs being in different genes for the Biofilter analysis (Fig 1 *).*

1 $$ \boldsymbol{reduced}:\kern0.75em y=\alpha +{\beta}_1(SNP1)+{\beta}_2(SNP2)+{\beta}_3(age)+{\beta}_4(BMI)+{\beta}_5(med.)+\kern0.5em {\beta}_6(T2D)+{\beta}_7(smoking)+{\beta}_8(sex)+{\beta}_{9-18}\left(PC1-PC10\right) $$

2 $$ \boldsymbol{full}:\kern0.75em y=\alpha +{\beta}_1(SNP1)+{\beta}_2(SNP2)+{\beta}_3(age)+{\beta}_4(BMI)+{\beta}_5(med.)+\kern0.5em {\beta}_6(T2D)+{\beta}_7(smoking)+{\beta}_8(sex)+{\beta}_{9-18}\left(PC1-PC10\right)+{\beta}_{19}\left(SNP{1}^{\ast }SNP2\right) $$

The full model consisted of the same SNP and covariate terms as the reduced model, but with an additional multiplicative interaction term for the SNP-SNP model. We generated “proxy” models by identifying SNPs in high linkage disequilibrium (LD) (r^2^ > 0.8) with model SNPs based on the HapMap European CEPH (CEU) population in 1000 Genomes Project Pilot 1 data (2010 release) using SNAP [21]. We generated a list of proxy SNP-SNP models using the SNPs in high LD with the original model SNPs to represent the original model from the discovery set. The purpose of these models was to capture signals in the replication data that may have been missed due to allele frequency differences between the discovery and replication cohorts. The original and proxy models from the discovery analysis were tested in each of the replication cohorts.

### Replication: Cohort descriptions

The top original and proxy models from the main effect filter and Biofilter analyses were tested in ten independent replication cohorts – BOSS/EHLS/BDES, CLEAR, eMERGE, EPIC, GIRaFH, KORA, LURIC, PROCARDIS, Whitehall II, and BWHHS. All of the replication cohorts, except the eMERGE datasets, were genotyped using the IBC array; therefore, many of the proxy models were not tested because many of the proxy SNPs are not on the IBC array.

The eMERGE network is a consortium of institutions with DNA from biorepositories linked to data from patient electronic medical records (EMR) [22]. The eMERGE set was genotyped with the Illumina660W GWAS platform and further imputed using 1000 Genomes project data, as described here [23]. The replication set consisted of data from the Marshfield Clinic, Northwestern University, Group Health Cooperative, Mayo Clinic, and Vanderbilt University. After quality control, the final eMERGE sample size was *n* = 7502 for all lipid traits. Details on quality control and phenotype extractions from the EMR can be found here [24].

The minimum variant and sample call rate threshold for all replication cohorts was 0.95 and 0.90, respectively. A Hardy-Weingberg equilibrium test *p*-value threshold of at least *p* < 1 × 10^−6^ was applied by each group. In each of the replication cohorts, population stratification and relatedness were assessed and adjusted for accordingly. All of the individuals in the replication cohorts were of European-American descent. The full details for the QC procedures can be found in the references provided for each replication cohort in Table 1.

### Replication: Quality control and statistical analyses

Replication analyses were performed in nine independent cohorts genotyped previously on the IBC array for a range of phenotypes including lipid levels [17] and the eMERGE cohort, which contained GWAS genotype data (Fig 1). For each of the ten cohorts, all of the models from the discovery analysis with LRT *p* < 0.001 and all of the corresponding proxy models were tested using the same statistical approach as for the discovery analysis (Eqs. 1 and 2). The same statistical approach was applied in the replication analysis as for the discovery analysis. We compiled the results to assess which SNP-SNP model signals replicated across respective cohorts. Significance of replication was assessed by correcting the likelihood ratio test *p*-value for the number of original (i.e. non-proxy) models tested and for the 10 replication cohorts. We also assessed how many of the 10 cohorts had significant replication for each of the models. These results were visualized using the program SynthesisView [25].

## Results

### Discovery and replication

Full results from the discovery analysis for all original models selected for replication testing can be found in Additional file 1: Table S1 and S2. The counts for the number of significant models that were identified and then tested in the replication cohort can be found in Fig. 1.

Significance in the replication cohort was estimated by using the number of original models tested in each study design (i.e. not counting the proxy models) and the number of replication cohorts (further divide by 10) to perform a Bonferroni-like correction equivalent to *p* = 0.05. For the MEF analyses, the number of original (non-proxy) models selected for testing are shown as: *lipid trait (model count, corrected p-value)* - HDL-C (156, *p* = 0.00003); LDL-C (160, *p* = 0.00003); TC (180, *p* = 0.00003); and TG (187, *p* = 0.00003). The respective counts for the Biofilter analysis were: HDL-C (22, *p* = 0.0002); LDL-C (22, *p* = 0.0002); TC (21, *p* = 0.0002); and TG (49, *p* = 0.0001). We then calculated the number of model signals that passed the respective thresholds in each cohort (i.e. if the original and proxy SNP-SNP models replicated for one LD signal then only one model signal was counted) (Fig. 1).

The models that passed the main effect filter and Biofilter replication significance threshold are shown in Tables 1 and 2, respectively. Results are shown for models with the same direction of effect as the discovery datasets and/or the lowest *p*-value, where replication was observed in more than one cohort. For the main effect filter analyses, more models passed the selected replication threshold. Also, a number of models showed similar results in more than one cohort. For HDL-C, 17 total models replicated with seven models observed to replicate in at least two cohorts. For LDL-C, two models replicated, both in at least two cohorts. For TC, replication occurred for one model in one cohort. For TG, 11 total models replicated, with four models replicating in at least two cohorts (Table 2). For the Biofilter analyses, results were replicated for the TG trait with two models passing the significance threshold in a single cohort (Table 3).

**Table 2:** Discovery and replication results for models passing replication thresholds for each lipid trait for main effect filter analysis

	Disc. Rank	SNP 1	SNP 2	Locus 1	Locus 2	Beta	LRT p	Rep. Beta	Rep. LRT p	Rep. Cohort^a^
HDL	1	rs12720918	rs4783961	*CETP*	*CETP*	−0.06	9.5 × 10^−20^	−0.07	3.0 × 10^−12^	P,W,L
	2	rs12720918	rs158477	*CETP*	*CETP*	−0.06	6.3 × 10^−16^	−0.07	2.9 × 10^−10^	P,W,L
	3	rs1864163	rs4783961	*CETP*	*CETP*	−0.06	4.5 × 10^−15^	−0.05	7.1 × 10^−7^	P,W,B
	5	rs1864163	rs158477	*CETP*	*CETP*	−0.06	1.3 × 10^−12^	−0.05	2.3 × 10^−8^	P
	6	rs12708967	rs820299	*CETP*	*CETP*	0.06	1.0 × 10^−11^	0.06	1.6 × 10^−6^	P,W,L
	7	rs1864163	rs4784744	*CETP*	*CETP*	0.05	2.6 × 10^−11^	0.06	5.2 × 10^−11^	P,W
	8	rs1800775	rs4783961	*CETP*	*CETP*	0.04	6.3 × 10^−11^	−0.08	2.4 × 10^−7^	B
	9	rs12708967	rs158477	*CETP*	*CETP*	−0.05	2.5 × 10^−10^	−0.06	1.1 × 10^−6^	P,W,L
	10	rs9939224	rs4783961	*CETP*	*CETP*	−0.05	2.5 × 10^−10^	−0.04	2.4 × 10^−7^	W,B
	12	rs1800775	rs158477	*CETP*	*CETP*	0.04	1.8 × 10^−8^	0.07	2.5 × 10^−6^	B
	13	rs9939224	rs478474	*CETP*	*CETP*	0.04	5.4 × 10^−7^	0.07	2.4 × 10^−10^	P
	17	rs1800775	rs4784744	*CETP*	*CETP*	−0.03	1.8 × 10^−6^	−0.05	9.7 × 10^−7^	W
	18	rs9939224	rs12447924	*CETP*	*CETP*	0.05	1.8 × 10^−6^	0.06	5.3 × 10^−7^	P
	38	rs7013777	rs9644636	*LPL*	*LPL*	−0.03	8.0 × 10^−5^	−0.04	7.5 × 10^−6^	W
	50	rs820299	rs8056954	*CETP*	*SLC12A3*	0.03	1.8 × 10^−4^	0.06	1.5 × 10^−5^	W
	66	rs12708967	rs4784744	*CETP*	*CETP*	0.03	3.0 × 10^−4^	0.05	2.4 × 10^−5^	P
	133	rs6586891	rs285	*LPL*	*LPL*	−0.02	7.9 × 10^−4^	−0.04	2.9 × 10^−5^	P
LDL	7	rs1531517	rs519113	*BCL3*	*PVRL2*	−0.16	7.9 × 10^−6^	−0.2	5.2 × 10^−6^	P,B
	70	rs4803766	rs157580	*PVRL2*	*TOMM40*	−0.06	3.4 × 10^−4^	−0.11	4.8 × 10^−7^	P,B
TC	33	rs11216129	rs10750097	*BUD13*	*APOA5*	−0.12	1.3 × 10^−4^	−0.22	1.4 × 10^−5^	W
TG	1	rs4938303	rs180327	*BUD13*	*BUD13*	0.09	1.2 × 10^−21^	0.08	9.5 × 10^−7^	P,W
	2	rs2075295	rs6589568	*BUD13*	*APOA4*	−0.10	4.4 × 10^−19^	−0.15	3.5 × 10^−15^	P,W
	3	rs180327	rs10750097	*BUD13*	*APOA5*	0.08	3.1 × 10^−14^	0.31	6.8 × 10^−9^	W,B
	4	rs180327	rs2075295	*BUD13*	*BUD13*	0.07	8.9 × 10^−13^	0.07	1.5 × 10^−5^	P
	5	rs180327	rs6589568	*BUD13*	*APOA4*	0.07	2.7 × 10^−10^	0.08	5.6 × 10^−6^	W
	6	rs11216129	rs10750097	*BUD13*	*APOA5*	−0.12	2.1 × 10^−9^	−0.12	2.6 × 10^−7^	W,P
	13	rs180327	rs618923	*BUD13*	*ZPR1*	−0.08	3.7 × 10^−7^	−0.08	1.0 × 10^−5^	W
	15	rs2075295	rs1263173	*BUD13*	*APOA4*	−0.08	2.1 × 10^−7^	−0.08	3.5 × 10^−6^	W
	19	rs486394	rs4938303	*BUD13*	*BUD13*	0.07	2.1 × 10^−6^	0.07	2.9 × 10^−5^	P
	49	rs2075295	rs10047459	*BUD13*	*APOA1*	−0.11	2.1 × 10^−5^	−0.11	6.9 × 10^−8^	W
	153	rs2075295	rs10750097	*BUD13*	*APOA5*	−0.09	2.1 × 10^−4^	−0.09	2.9 × 10^−6^	W

*LRT* likelihood ratio test. ^a^See Table 1 for details on cohorts

**Table 3:** Models that passed replications threshold for the TG trait for Biofilter analysis

	Disc. Rank	SNP 1	SNP 2	Locus 1	Locus 2	Beta	LRT p	Rep. Beta	Rep. LRT p	Rep. Cohort^a^
TG	9	rs11216162	rs1263173	*SIK3*	*APOA4*	−0.05	5.5 × 10^−5^	−0.08	5.5 × 10^−5^	P
	44	rs625145	rs1263173	*SIK3*	*APOA4*	−0.04	6.8 × 10^−5^	−0.07	6.8 × 10^−5^	P

No models passed replication for HDL-C, LDL-C, or TC, *LRT* likelihood ratio test. ^a^ See Table 1 for details on cohorts

**Fig. 2:**
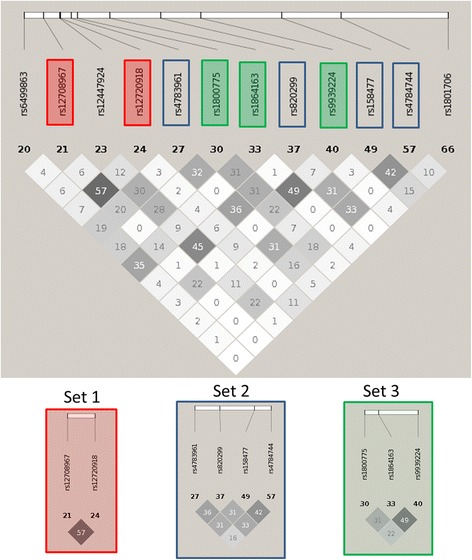
Pairwise r^2^ values for SNPs in top models for main effect filtering (MEF) analysis of HDL-C levels. The numbers in the boxes are r^2^ values and *darker shading* indicates higher LD. The numbers below the SNPs are an indicator of location in this region. Correlation patterns indicate three sets of SNPs and two interaction signals based on replication results (Set 1 x Set 2 and Set 2 x Set 3)

Although we performed LD pruning prior to the interaction analyses, moderate LD remained with r^2^ < 0.6. This resulted in residual correlation in the top replicating models, and separate models may actually represent a single interaction signal. Additionally, all of the replicating models contained two SNPs in the same gene/region. To assess this, we looked at the pairwise r^2^values amongst all SNPs in the top replication models. The goal was to identify independent replication signals and to ensure that the interaction signals are not being inflated by LD between model SNPs. For the main effect filter analysis of HDL-C, we identified three sets of moderately correlated SNPs and two interaction signals (*Set 1 x Set 2* and *Set 2 x Set 3*), as shown in Fig. 2. No correlation (r^2^ > 0.1) was observed in our data between SNPs in the same replication model.

**Fig. 3:**
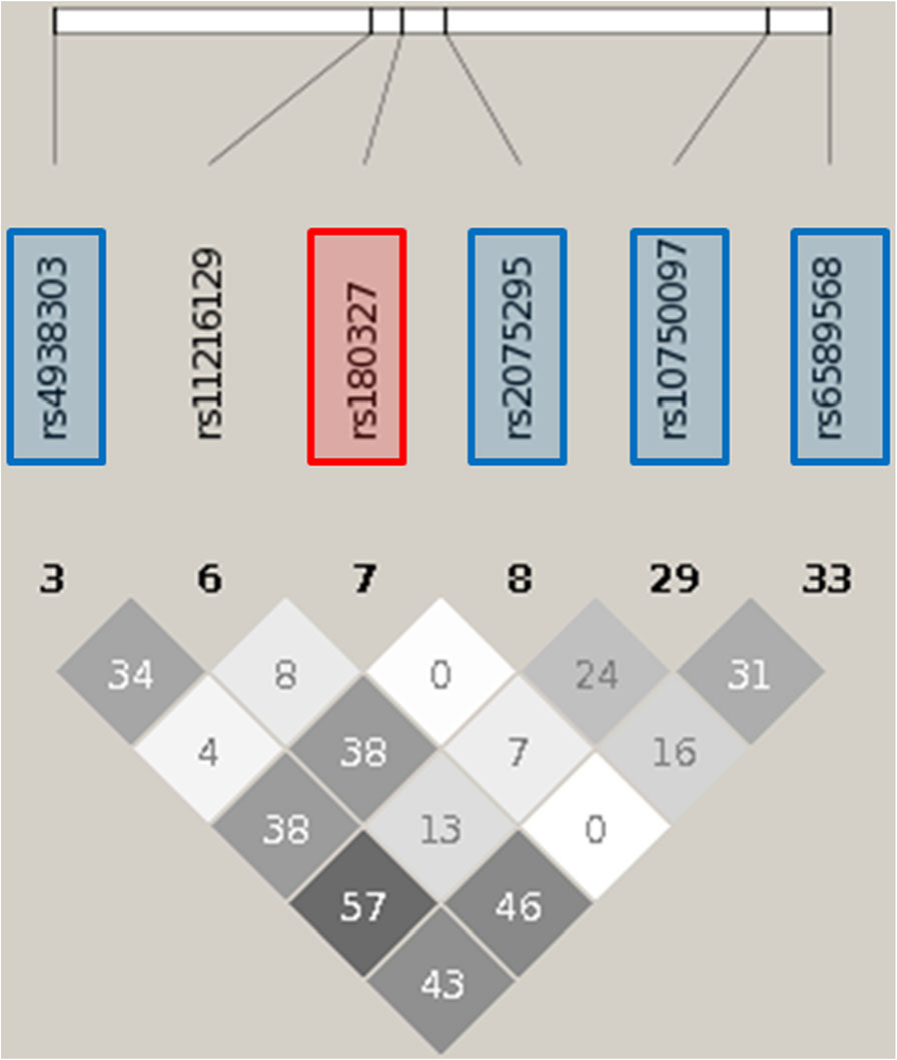
Pairwise r^2^ values for SNPs in top models for the main effect filtering (MEF) replication analysis of plasma triglyceride (TG) levels. The numbers in the boxes are r^2^ values and *darker shading* indicates higher LD. The numbers below the SNPs are an indicator of location in this region. Correlation patterns indicate a single signal representing an interaction between rs180327 (or a correlated functional variant) and the other variants for the four models that include this SNP

For the main effect filter analyses of TG, the signals that replicated were in a similar region on chromosome 11. This region includes several genes with strong main effects on TG levels, including *APOA4*, *APOA5*, *APOC3*, *SIK3*, and *BUD13*. There are complex patterns of moderate to strong LD in this region, and thus bona fide “independent” signals are challenging to delineate. However, for the main effect filter analyses of TG, one SNP (rs180327) appeared in two of the four models that replicated in more than one cohort. Moderate correlation exists between most of the other SNPs except for rs180327 (Fig. 3). This suggests a single signal representing an interaction between rs180327 (or a correlated functional variant) and the other variants for the four models that include this SNP. For the main effect filter analysis of LDL, two models replicated in more than one cohort. While the SNPs from the two models are in a similar region on chromosome 19 encompassing genes such as *APOE, BCL3, PVRL2*, and *TOMM40*, these appear to consist of two separate interaction signals. No models replicated in HDL-C, LDL-C, or TC for the Biofilter analyses.

**Fig. 4:**
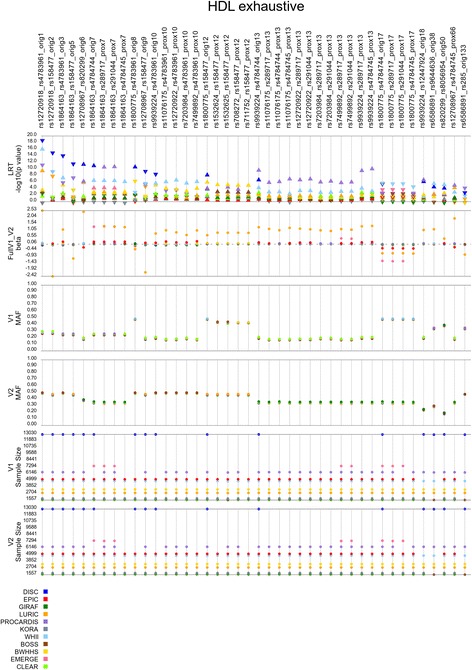
Results for the main effect filter (MEF) analysis of HDL-C. Showing results for the models that passed the replication threshold of *p* < 3.0 × 10–5. Orig# and prox# designate models that were identified in the discovery cohort and those identified via proxy (i.e. both SNPs in high LD with SNPs from orig. Models), respectively. V1 and V2 are the two SNPs in the model; *arrow* in likelihood ratio test (LRT) row denotes direction of effect

**Fig. 5:**
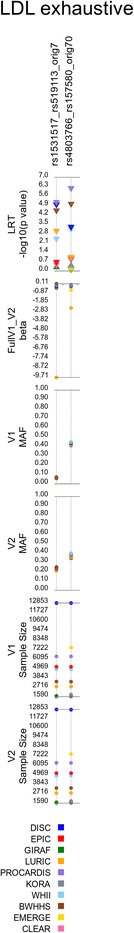
Results for the main effect filter (MEF) analysis of LDL-C. Showing results for the models that passed the replication threshold of *p* < 3.0 × 10–5. Orig# and prox# designate models that were identified in the discovery cohort and those identified via proxy (i.e. both SNPs in high LD with SNPs from orig. Models), respectively. V1 and V2 are the two SNPs in the model; *arrow* in likelihood ratio test (LRT) row denotes direction of effect

**Fig. 6:**
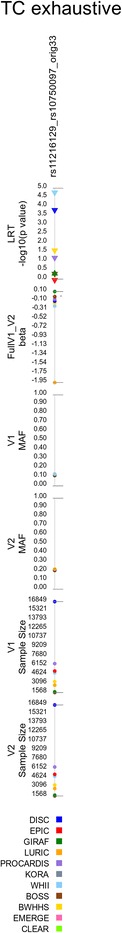
Results for the main effect filter (MEF) analysis of TC. Showing results for the models that passed the replication threshold of *p* < 3.0 × 10–5. Orig# and prox# designate models that were identified in the discovery cohort and those identified via proxy (i.e. both SNPs in high LD with SNPs from orig. Models), respectively. V1 and V2 are the two SNPs in the model; arrow in likelihood ratio test (LRT) row denotes direction of effect

**Fig. 7:**
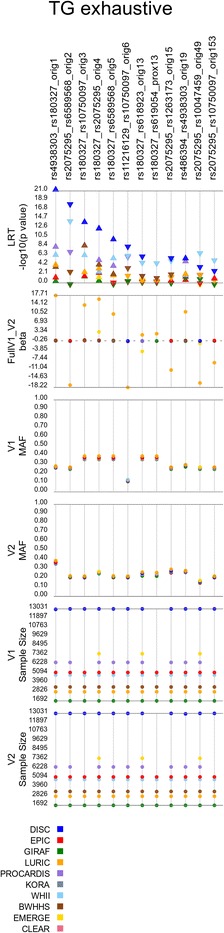
Results for the main effect filter (MEF) analysis of TG. Showing results for the models that passed the replication threshold of *p* < 3.0 × 10–5. Orig# and prox# designate models that were identified in the discovery cohort and those identified via proxy (i.e. both SNPs in high LD with SNPs from orig. Models), respectively. V1 and V2 are the two SNPs in the model; arrow in likelihood ratio test (LRT) row denotes direction of effect

**Fig. 8:**
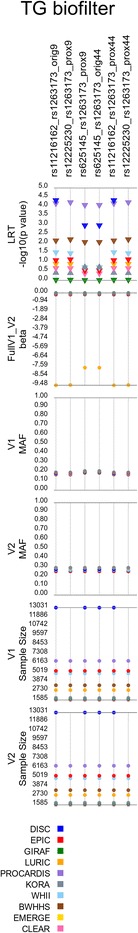
Results for the Biofilter analysis of TG. Showing results for the models that passed the replication threshold of *p* < 3.0 × 10–5. Orig# and prox# designate models that were identified in the discovery cohort and those identified via proxy (i.e. both SNPs in high LD with SNPs from orig. Models), respectively. V1 and V2 are the two SNPs in the model; arrow in likelihood ratio test (LRT) row denotes direction of effect

To further summarize the replication analyses, we plotted the compiled results to view all of the cohorts’ results simultaneously for each of the analyses with significant replication (Figs. 4, 5, 6, 7 and 8). In these figures, we show the models that replicated at the aforementioned thresholds. As some of the replications are in proxy models (not the original discovery model), we show the lead significant result for the each replicating model.

## Discussion

For this study, we used two different filtering pipelines to test for SNP-SNP interactions that are associated with four plasma lipid level traits: LDL-C, HDL-C, TC, and TG plasma levels. We tested these models in a large discovery cohort and then tested the top models in ten replication sets. Models signals passed the replication threshold for each of the lipid traits in the main effect filter analysis and for TG in the Biofilter analysis. As expected, replication of the observed association was found to be dependent on the size of the replication cohorts. Also, more models replicated in the main effect filter analysis, which may indicate a statistical bias due to strong main effects. However, the interaction signals appear robust, considering the number of models tested, indicating that this is unlikely the sole driver of these significant interactions.

Genetic interactions are often described as gene-gene interactions, and are usually studied by specifically looking for variants in different genes that could be indicating novel pathways (e.g. protein-protein interactions that have not been previously identified using genetic data). However, intergenic interactions, such as those that we observed in this study, should not be ignored, as they may contribute to a substantial proportion of the genetic architecture. Our top replicating models for HDL-C consisted of two SNPs in CETP. Many of these model replicated across cohorts with the top replication *p*-value for the likelihood ratio test being 3.0 × 10–12 (Table 2 and Additional file 1: Table S1). LD patterns suggest that there are three independent sets of SNPs that represent many of the top models for the CETP-HDL associations. Further, many of these SNPs are in the promoter region of CETP. Most notably, a previous study identified a functional interaction between two of the SNPs in one of our top models (model 9: rs4783961 and rs1800775) that resulted in changes in CETP promoter activity [26]. As discussed in this study, this could be explained by shared transcription factors that may result in non-linear changes in CETP and HDL-C levels when the variants occur together. These results provide further support for studying intergenic non-linear effects and that they could be important for both accurate phenotype prediction and for understanding the function behind why specific variants in this gene have certain effects on HDL-C levels.

Due to the complex nature of estimating heritability, we focus on how our results contribute to understanding the genetic architecture and biological underpinnings of lipid traits. First, the estimated heritability of lipid traits has a relatively wide range (40–60%). There is also high variability in results that can come from methods that calculate overall heritability. A recent study found that for certain models, the estimate is extremely inflated and potentially not reliable [12]. Furthermore, because we are studying genetic interactions, reliably calculating the overall contribution to trait variation becomes even more complicated, and many methods are not designed to accurately generate these estimates.

In our study, we can see the difference in R2 for the full versus reduced model (Diff|Rsq column in Additional file 1: Table S1 and S2) is usually about 0.001. Even though this is much smaller than the R2 for the reduced model, which does not include the interaction term, it would be inaccurate to conclude that the interaction term is not contributing to the underlying genetic variation for a number of reasons. Firstly, the reduced model includes the contribution of highly relevant clinical and environmental variable (e.g. smoking status, medication, BMI, sex). Secondly, we are calculating this estimate from a very specific interaction model that assumes the interaction is multiplicative and that the effect of minor alleles is additive. While this model is robust to some interactions that don’t meet these assumptions [13], the estimates themselves could be under-estimated (or over-estimated). As we are not the first group to look for genetic interactions amongst lipid traits, it will be very important for a future study to be done that takes into account all of the identified main and interaction effects to assess and compare the contribution of each to trait variability. However, this is outside the scope of our current manuscript.

Many questions remain to be answered in regards to a gold-standard genome-wide or candidate-loci interaction analysis protocol. For example, the overwhelming majority of our replicating interaction models were in the same gene. This is most likely due to the fact that our variants were genotyped using a gene-centric chip with genes that are known to have effects on cardiovascular-related phenotypes, like the lipid levels we analyzed in this study. A chip that had more extensive coverage outside of these genes may have identified more interactions between functionally different parts of the genome. However, our focused analysis did allow us to efficiently test two unique filtering pipelines for a more hypothesis-driven approach. These filtering approaches each have their own strengths and weaknesses. The Biofilter 2.0 analysis, which created gene-gene models based on current biological knowledge, allows for clearer interpretations as the models make biological sense. However, it inhibits the discovery of interactions in regions where biological knowledge is limited. The main effect filter analysis is more robust to discovering interactions that deviate from regions of current biological focus than the Biofilter pipeline in this particular dataset. However, if the main effects of the true interaction model are non-existent (i.e. purely epistatic models where each of the SNPs in the interaction model are not significant alone), the main effect filter pipeline would not detect such effects. Also, as our results possibly indicate, strong main effects may create inflated interaction signals. A more appropriate filtering pipeline may use a main effect filter – Biofilter hybrid approach. Another possible filtering mechanism may be one that ranks variables based on potential main and interaction effects simultaneously. Some machine learning methods, such as Random Forests (RF) and artificial neural networks (ANN), are currently being tested for this approach [27].

## Additional file

Additional file 1: Table S1. Discovery results for all models that passed replication thresholds for MEF analysis. Column header definitions provided at the end. **Table S2.** Discovery results for all models that passed replication thresholds for Biofilter analysis. Column header definitions provided at the end. (PDF 1649 kb)
