# Supplementary material for: Discovery and replication of SNP-SNP interactions for quantitative lipid traits in over 60,000 individuals
Source: BioData Min. 2017 Jul 24;10:25. doi: 10.1186/s13040-017-0145-5 (PMC5525436; doi:10.1186/s13040-017-0145-5)
Supplement: Additional file 1: Table S1. — Discovery results for all models that passed replication thresholds for MEF analysis. Column header definitions provided at the end. Table S2. Discovery results for all models that passed replication thresholds for Biofilter analysis. Column header definitions provided at the end. (PDF 1649 kb) [file 13040_2017_145_MOESM1_ESM.pdf]

S1 Table: Discovery results for all models that passed replication thresholds for MEF analysis. Column header definitions provided at the end.

Lipid	V1	V2	V1|chr:bp	V2|chr:bp	V1|MAF	V2|MAF	V1|Beta	V1|pval	V2|Beta	V2|pval	Int|Beta	Full|Rsq	Diff|Rsq	lrt|pval
HDL	rs12720918	rs4783961	16:55551713	16:55552395	0.278	0.496	0.013	0.15178	0.085	1.17E-47	-0.064	0.299	0.004	9.52E-20
HDL	rs12720918	rs158477	16:55551713	16:55565111	0.278	0.473	0.006	0.46461	0.044	5.62E-14	-0.057	0.291	0.004	5.93E-16
HDL	rs1864163	rs4783961	16:55554734	16:55552395	0.253	0.496	-0.011	0.1886	0.067	1.41E-31	-0.056	0.300	0.003	4.47E-15
HDL	rs1864163	rs820299	16:55554734	16:55557785	0.253	0.383	-0.111	8.61E-51	-0.059	1.29E-24	0.057	0.298	0.003	2.17E-14
HDL	rs1864163	rs158477	16:55554734	16:55565111	0.253	0.473	-0.019	0.02952	0.050	5.61E-18	-0.051	0.296	0.003	1.33E-12
HDL	rs12708967	rs820299	16:55550712	16:55557785	0.182	0.383	-0.102	1.63E-35	-0.051	5.96E-21	0.058	0.293	0.003	1.03E-11
HDL	rs1864163	rs4784744	16:55554734	16:55568686	0.253	0.341	-0.101	1.37E-41	-0.054	9.21E-19	0.049	0.296	0.002	2.63E-11
HDL	rs1800775	rs4783961	16:55552737	16:55552395	0.485	0.496	0.030	0.00031	-0.038	3.03E-06	0.041	0.299	0.002	6.34E-11
HDL	rs12708967	rs158477	16:55550712	16:55565111	0.182	0.473	-0.010	0.31185	0.040	1.63E-13	-0.051	0.292	0.002	2.46E-10
HDL	rs9939224	rs4783961	16:55560233	16:55552395	0.204	0.496	-0.027	0.00569	0.064	2.81E-32	-0.046	0.299	0.002	1.85E-09
HDL	rs12720918	rs820299	16:55551713	16:55557785	0.278	0.383	-0.084	1.75E-33	-0.060	3.06E-24	0.043	0.293	0.002	8.91E-09
HDL	rs1800775	rs158477	16:55552737	16:55565111	0.485	0.473	0.039	2.28E-07	-0.035	4.65E-06	0.035	0.299	0.002	1.76E-08
HDL	rs9939224	rs4784744	16:55560233	16:55568686	0.204	0.341	-0.100	1.49E-38	-0.051	7.36E-20	0.041	0.296	0.001	5.42E-07
HDL	rs12708967	rs9923854	16:55550712	16:55574503	0.182	0.103	-0.075	1.32E-30	0.001	0.94559	0.060	0.290	0.001	8.18E-07
HDL	rs9939224	rs158477	16:55560233	16:55565111	0.204	0.473	-0.036	0.00045	0.047	2.06E-17	-0.039	0.296	0.001	8.57E-07
HDL	rs1800775	rs820299	16:55552737	16:55557785	0.485	0.383	0.105	2.29E-52	0.047	3.01E-10	-0.032	0.299	0.001	1.33E-06
HDL	rs1800775	rs4784744	16:55552737	16:55568686	0.485	0.341	0.091	2.90E-46	0.026	0.00038	-0.033	0.298	0.001	1.75E-06
HDL	rs9939224	rs12447924	16:55560233	16:55551693	0.204	0.238	-0.094	4.96E-44	-0.052	1.82E-17	0.047	0.296	0.001	1.81E-06
HDL	rs2952101	rs12976922	23:14768515	19:60562163	0.401	0.134	0.004	0.30064	0.033	0.00023	-0.036	0.284	0.001	2.95E-06
HDL	rs6499863	rs158477	16:55549518	16:55565111	0.164	0.473	0.001	0.90694	0.028	7.15E-08	-0.040	0.286	0.001	3.67E-06
HDL	rs1683564	rs2025766	19:810214	9:112729248	0.342	0.138	-0.015	0.0056	-0.034	0.0003	0.043	0.285	0.001	5.16E-06
HDL	rs12708967	rs7204044	16:55550712	16:55500210	0.182	0.24	-0.039	6.63E-08	0.028	6.60E-06	-0.043	0.290	0.001	1.35E-05
HDL	rs12708967	rs4567697	16:55550712	16:55503120	0.182	0.456	-0.027	0.00284	0.024	8.34E-06	-0.036	0.290	0.001	1.90E-05
HDL	rs12720918	rs1801706	16:55551713	16:55575163	0.278	0.177	-0.072	8.39E-29	0.007	0.46	0.036	0.290	0.001	2.25E-05
HDL	rs1531517	rs4841324	19:49934013	8:10238942	0.066	0.093	0.005	0.63936	-0.007	0.40257	0.094	0.284	0.001	2.34E-05
HDL	rs9939224	rs820299	16:55560233	16:55557785	0.204	0.383	-0.106	4.89E-45	-0.056	4.29E-25	0.038	0.298	0.001	2.85E-05
HDL	rs16982735	rs2102928	19:56686663	17:35506754	0.235	0.317	0.024	0.00119	0.026	2.83E-05	-0.033	0.283	0.001	3.40E-05
HDL	rs2697920	rs1869138	11:47327183	15:56566331	0.337	0.096	0.017	0.00114	0.044	4.34E-05	-0.047	0.284	0.001	3.60E-05
HDL	rs6499863	rs820299	16:55549518	16:55557785	0.164	0.383	-0.062	1.18E-09	-0.033	1.54E-09	0.035	0.286	0.001	3.88E-05
HDL	rs4784744	rs6499863	16:55568686	16:55549518	0.341	0.164	-0.041	8.33E-14	-0.057	1.99E-10	0.035	0.287	0.001	3.88E-05
HDL	rs12708967	rs8056954	16:55550712	16:55487730	0.182	0.178	-0.074	1.39E-24	-0.032	1.17E-05	0.041	0.289	0.001	4.17E-05
HDL	rs572410	rs1531517	15:56528676	19:49934013	0.238	0.066	0.024	9.64E-06	-0.009	0.44533	0.060	0.286	0.001	4.29E-05
HDL	rs11719375	rs2065412	3:10681597	9:106638561	0.189	0.405	0.013	0.11979	0.005	0.33469	-0.033	0.284	0.001	5.07E-05
HDL	rs6584353	rs2065412	10:101977045	9:106638561	0.066	0.405	0.025	0.07613	-0.001	0.87242	-0.052	0.284	0.001	5.18E-05
HDL	rs12708967	rs1801706	16:55550712	16:55575163	0.182	0.177	-0.073	1.72E-26	-0.002	0.74986	0.042	0.289	0.001	6.66E-05
HDL	rs2287499	rs6745266	17:7532893	2:239858512	0.117	0.128	-0.005	0.49653	0.013	0.08474	-0.059	0.284	0.001	7.02E-05
HDL	rs650326	rs6036157	3:137520397	20:22526415	0.404	0.067	0.002	0.72791	0.052	0.00013	-0.051	0.284	0.001	7.28E-05
HDL	rs6586891	rs9644636	8:19958878	8:19869176	0.347	0.287	-0.046	1.61E-13	-0.041	1.59E-09	0.030	0.286	0.001	7.53E-05
HDL	rs7013777	rs9644636	8:19922636	8:19869176	0.46	0.287	0.044	1.22E-12	0.001	0.89995	-0.028	0.286	0.001	7.99E-05
HDL	rs650326	rs4796751	3:137520397	17:37381063	0.404	0.149	0.005	0.31342	0.020	0.03722	-0.035	0.284	0.001	9.37E-05
HDL	rs4938303	rs180327	11:116090197	11:116128869	0.271	0.371	-0.002	0.76548	0.002	0.70601	-0.028	0.285	0.001	9.96E-05
HDL	rs2418736	rs4144739	16:66712363	8:32089597	0.182	0.499	-0.007	0.46787	-0.005	0.34144	0.032	0.284	0.001	0.0001
HDL	rs12708967	rs289716	16:55550712	16:55566877	0.182	0.316	-0.073	9.03E-22	0.022	8.05E-05	0.035	0.292	0.001	0.00011
HDL	rs9295131	rs3811647	6:161056284	3:134966719	0.305	0.337	0.004	0.57554	0.011	0.09229	-0.028	0.284	0.001	0.00011
HDL	rs3803501	rs6036157	15:61139145	20:22526415	0.428	0.067	-0.014	0.00261	0.051	0.00022	-0.049	0.285	0.001	0.00013
HDL	rs1043908	rs339538	1:228483917	13:59473699	0.115	0.358	0.049	1.05E-06	0.004	0.49471	-0.038	0.284	0.001	0.00013
HDL	rs10099160	rs7204044	8:19866095	16:55500210	0.235	0.24	0.008	0.23015	0.031	2.47E-06	-0.033	0.284	0.001	0.00014
HDL	rs842207	rs4841324	12:42751537	8:10238942	0.144	0.093	0.003	0.6717	-0.012	0.19281	0.058	0.283	0.001	0.00015
HDL	rs7013777	rs285	8:19922636	8:19859469	0.46	0.476	-0.002	0.73959	-0.001	0.94231	0.024	0.285	0.001	0.00017
HDL	rs820299	rs8056954	16:55557785	16:55487730	0.383	0.178	-0.036	4.99E-11	-0.046	4.44E-07	0.031	0.286	0.001	0.00017
HDL	rs157580	rs1487562	19:50087106	10:45248828	0.389	0.183	-0.001	0.83041	-0.014	0.1074	0.031	0.284	0.001	0.00018
HDL	rs1998064	rs11706810	1:228353751	3:161642615	0.247	0.483	-0.018	0.03598	-0.020	0.00048	0.027	0.284	0.001	0.00018
HDL	rs4301628	rs12507396	1:113294385	4:156348494	0.33	0.117	0.015	0.00457	0.016	0.107	-0.039	0.283	0.001	0.0002
HDL	rs4245232	rs3204635	18:45340996	12:55923860	0.191	0.269	0.009	0.25096	0.004	0.46879	-0.033	0.284	0.001	0.0002

S1 Table, cont'd

Lipid	V1	V2	V1|chr:bp	V2|chr:bp	V1|MAF	V2|MAF	V1|Beta	V1|pval	V2|Beta	V2|pval	Int|Beta	Full|Rsq	Diff|Rsq	lrt|pval
HDL	rs2472449	rs6584353	9:106644018	10:101977045	0.342	0.066	0.009	0.07791	-0.053	4.86E-05	0.051	0.284	0.001	0.00022
HDL	rs1019179	rs282176	7:38185501	1:26771543	0.235	0.274	0.027	0.0001	0.025	8.62E-05	-0.031	0.284	0.001	0.00022
HDL	rs11986942	rs7459730	8:19911725	8:9034170	0.309	0.155	0.047	3.35E-17	0.008	0.34142	-0.035	0.287	0.001	0.00025
HDL	rs1131877	rs613084	14:102411802	11:68359733	0.237	0.35	-0.027	0.00042	-0.016	0.00578	0.028	0.283	0.001	0.00025
HDL	rs247615	rs820299	16:55542264	16:55557785	0.222	0.383	-0.043	4.21E-08	-0.039	6.30E-12	0.029	0.286	0.001	0.00025
HDL	rs486394	rs3849591	11:116031532	4:46063398	0.289	0.171	-0.006	0.29886	0.033	3.46E-05	-0.034	0.284	0.001	0.00025
HDL	rs618923	rs1451678	11:116159369	11:14738197	0.251	0.489	-0.010	0.27573	-0.007	0.20514	0.027	0.284	0.001	0.00026
HDL	rs2777799	rs9900564	9:106598880	17:73889053	0.115	0.348	0.018	0.07563	-0.002	0.72403	-0.037	0.284	0.001	0.00027
HDL	rs1869138	rs29228	15:56566331	6:29731718	0.096	0.198	0.032	0.00054	0.000	0.99172	-0.050	0.283	0.001	0.00028
HDL	rs7525142	rs12460089	1:238809528	19:7141716	0.22	0.372	0.015	0.06215	0.002	0.78293	-0.029	0.284	0.001	0.0003
HDL	rs1683564	rs2239181	19:810214	12:46542216	0.342	0.106	0.005	0.32038	0.015	0.16055	-0.039	0.284	0.001	0.0003
HDL	rs12708967	rs4784744	16:55550712	16:55568686	0.182	0.341	-0.079	1.13E-22	-0.044	5.46E-15	0.031	0.292	0.001	0.0003
HDL	rs3764261	rs2482419	16:55550825	9:106643863	0.32	0.106	0.079	6.50E-51	-0.005	0.61808	0.039	0.302	0.001	0.00031
HDL	rs8093249	rs12575157	18:45351396	11:47229322	0.154	0.14	0.001	0.87246	0.009	0.20908	-0.046	0.283	0.001	0.00031
HDL	rs2482419	rs12358632	9:106643863	10:84101439	0.106	0.077	0.031	5.57E-05	0.006	0.50008	-0.068	0.284	0.001	0.00031
HDL	rs2980885	rs11706810	8:126543488	3:161642615	0.231	0.483	0.014	0.11467	0.006	0.30448	-0.026	0.284	0.001	0.00032
HDL	rs611841	rs2131127	1:228376104	3:149906833	0.157	0.325	-0.007	0.42495	0.030	8.71E-08	-0.033	0.285	0.001	0.00032
HDL	rs583219	rs10497434	11:116228381	2:175724086	0.053	0.065	0.010	0.35428	0.011	0.2314	0.096	0.284	0.001	0.00032
HDL	rs767067	rs3820809	16:3236340	2:238309774	0.061	0.093	0.007	0.49938	-0.005	0.55997	-0.082	0.283	0.001	0.00033
HDL	rs1487562	rs2522133	10:45248828	12:119239842	0.183	0.291	-0.009	0.26586	-0.014	0.01665	0.032	0.283	0.001	0.00034
HDL	rs236996	rs10800064	4:88224227	1:163215369	0.399	0.497	-0.011	0.16233	-0.015	0.02716	0.023	0.284	0.001	0.00034
HDL	rs2290146	rs3960965	11:47310074	19:58481195	0.172	0.262	0.000	0.96889	-0.017	0.00653	0.034	0.284	0.001	0.00035
HDL	rs12134960	rs3811647	1:195276131	3:134966719	0.154	0.337	-0.007	0.4219	-0.017	0.0023	0.033	0.283	0.001	0.00035
HDL	rs572410	rs38902	15:56528676	7:116676954	0.238	0.476	0.057	3.92E-11	0.028	8.21E-07	-0.026	0.286	0.001	0.00035
HDL	rs2943634	rs6705820	2:226776324	2:69827241	0.33	0.486	-0.011	0.17102	-0.010	0.10953	0.024	0.283	0.001	0.00035
HDL	rs873985	rs12827659	3:50626755	12:26236473	0.159	0.056	0.003	0.69634	0.030	0.00917	-0.067	0.283	0.001	0.00037
HDL	rs6499863	rs2066716	16:55549518	9:106608526	0.164	0.088	-0.043	2.66E-11	0.000	0.97147	0.053	0.285	0.001	0.00037
HDL	rs263	rs783149	8:19857092	6:161008908	0.179	0.166	0.050	2.59E-13	-0.002	0.76813	-0.040	0.286	0.001	0.00038
HDL	rs17639446	rs11084083	17:45628902	19:56701372	0.115	0.242	-0.007	0.41204	-0.002	0.69066	0.040	0.283	0.001	0.00038
HDL	rs611841	rs10488	1:228376104	11:102173232	0.157	0.061	-0.037	1.68E-08	-0.022	0.04209	0.063	0.284	0.001	0.00041
HDL	rs10099160	rs4567697	8:19866095	16:55503120	0.235	0.456	0.016	0.05947	0.027	1.80E-06	-0.026	0.284	0.001	0.00042
HDL	rs663214	rs4803766	15:41901074	19:50063008	0.282	0.43	0.016	0.04425	0.019	0.00143	-0.025	0.284	0.001	0.00042
HDL	rs12720918	rs289716	16:55551713	16:55566877	0.278	0.316	-0.070	7.14E-23	0.030	3.21E-06	0.026	0.293	0.001	0.00043
HDL	rs158477	rs8056954	16:55565111	16:55487730	0.473	0.178	0.029	5.86E-08	0.005	0.58286	-0.029	0.285	0.001	0.00045
HDL	rs572410	rs6947329	15:56528676	7:116730371	0.238	0.395	0.053	1.33E-11	0.024	2.69E-05	-0.026	0.286	0.001	0.00045
HDL	rs9939224	rs776385	16:55560233	8:31856167	0.204	0.295	-0.054	6.77E-13	0.012	0.0461	-0.030	0.293	0.001	0.00049
HDL	rs3761685	rs10751382	1:158351267	10:45256365	0.128	0.32	-0.004	0.64802	-0.011	0.0341	0.036	0.284	0.001	0.0005
HDL	rs13326165	rs4149336	3:52507158	9:106590460	0.192	0.181	-0.001	0.91547	-0.001	0.88381	0.037	0.284	0.001	0.00051
HDL	rs6420185	rs11187519	8:144374446	10:95329965	0.392	0.122	0.016	0.00163	0.016	0.12512	-0.034	0.283	0.001	0.00051
HDL	rs2001945	rs260815	8:126547160	11:103423950	0.481	0.347	-0.004	0.4979	0.025	0.00187	-0.023	0.284	0.001	0.00052
HDL	rs3761685	rs2494743	1:158351267	14:104322765	0.128	0.096	0.007	0.35149	0.008	0.36142	0.055	0.284	0.001	0.00053
HDL	rs2980884	rs11706810	8:126543538	3:161642615	0.37	0.483	0.009	0.27561	0.010	0.11872	-0.023	0.284	0.001	0.00053
HDL	rs17608302	rs6705820	10:121119157	2:69827241	0.224	0.486	-0.041	4.87E-06	-0.006	0.30652	0.026	0.284	0.001	0.00054
HDL	rs4810479	rs8100151	20:43978455	19:38496239	0.251	0.142	-0.014	0.01679	0.010	0.23442	-0.036	0.284	0.001	0.00055
HDL	rs202720	rs3812265	11:49148927	7:134699344	0.227	0.243	-0.004	0.52107	-0.021	0.001	0.029	0.283	0.001	0.00055
HDL	rs17269264	rs1481031	15:56526052	18:59003065	0.482	0.328	0.033	9.70E-08	0.032	4.67E-05	-0.023	0.284	0.001	0.00056
HDL	rs3847305	rs4973605	9:106697074	2:233670290	0.145	0.284	0.000	0.99765	0.015	0.00805	-0.034	0.284	0.001	0.00056
HDL	rs3803501	rs11899823	15:61139145	2:43655216	0.428	0.333	-0.036	1.33E-08	-0.023	0.00157	0.023	0.284	0.001	0.00058
HDL	rs17364665	rs10488	15:46672121	11:102173232	0.072	0.061	0.002	0.83678	-0.014	0.15285	0.088	0.283	0.001	0.00058
HDL	rs1043908	rs4788423	1:228483917	16:23926364	0.115	0.364	-0.004	0.69435	-0.008	0.11465	0.035	0.283	0.001	0.0006
HDL	rs12720918	rs7204044	16:55551713	16:55500210	0.278	0.24	-0.032	1.49E-07	0.024	0.00041	-0.030	0.288	0.001	0.00061
HDL	rs2980883	rs12358632	8:126543587	10:84101439	0.153	0.077	-0.024	0.00028	-0.025	0.00917	0.057	0.284	0.001	0.00063
HDL	rs4953023	rs11076696	2:43927504	16:87267489	0.069	0.339	-0.024	0.04793	0.003	0.54134	0.044	0.283	0.001	0.00064
HDL	rs2287499	rs1047307	17:7532893	2:182109997	0.117	0.348	0.004	0.69796	0.002	0.70131	-0.035	0.284	0.001	0.00064

S1 Table, cont'd

Lipid	V1	V2	V1|chr:bp	V2|chr:bp	V1|MAF	V2|MAF	V1|Beta	V1|pval	V2|Beta	V2|pval	Int|Beta	Full|Rsq	Diff|Rsq	lrt|pval
HDL	rs253	rs1131877	8:19855697	14:102411802	0.451	0.237	0.021	0.000195	0.016	0.056464	-0.026	0.283	0.001	0.000646
HDL	rs10438303	rs3746972	15:41803709	21:45152263	0.099	0.179	-0.001	0.926729	0.014	0.034536	-0.047	0.283	0.001	0.000653
HDL	rs9295131	rs6803163	6:161056284	3:169410950	0.305	0.054	-0.009	0.072452	0.036	0.007567	-0.05	0.284	0.001	0.000657
HDL	rs12454712	rs4710958	18:58996864	6:21018982	0.38	0.294	-0.012	0.057036	-0.022	0.014713	0.024	0.283	0.001	0.000657
HDL	rs2517955	rs730079	17:35097207	19:18764495	0.344	0.381	-0.033	3.34E-06	-0.018	0.005772	0.023	0.284	0.001	0.000658
HDL	rs6031587	rs12321904	20:42471663	12:108473611	0.07	0.478	-0.049	0.000687	-0.018	0.000161	0.042	0.284	0.001	0.000661
HDL	rs3857080	rs12629751	4:149355890	3:12374407	0.102	0.086	0.01	0.218288	-0.029	0.001394	0.063	0.284	0.001	0.000676
HDL	rs405509	rs873985	19:50100676	3:50626755	0.486	0.159	-0.023	6.54E-06	-0.034	0.001142	0.029	0.284	0.001	0.000683
HDL	rs1801700	rs10497520	2:21099318	2:179353100	0.051	0.133	-0.011	0.340415	0.001	0.913643	-0.073	0.284	0.001	0.000685
HDL	rs9900564	rs6745266	17:73889053	2:239858512	0.348	0.128	-0.019	0.000276	-0.024	0.014881	0.034	0.284	0.001	0.000688
HDL	rs799166	rs11084083	7:72689868	19:56701372	0.126	0.242	0.039	5.70E-06	0.017	0.004868	-0.037	0.284	0.001	0.000688
HDL	rs4100654	rs2952101	9:106709062	23:14768515	0.093	0.401	-0.045	1.50E-05	-0.011	0.006856	0.03	0.284	0.001	0.000691
HDL	rs4245232	rs3960965	18:45340996	19:58481195	0.191	0.262	0.007	0.349767	0.008	0.206439	-0.031	0.283	0.001	0.000697
HDL	rs7394871	rs2715554	11:61409090	17:35742696	0.053	0.153	-0.045	0.000124	-0.024	0.000228	0.066	0.284	0.001	0.0007
HDL	rs2482424	rs1554527	9:106705572	15:36432881	0.138	0.106	-0.011	0.140625	-0.033	8.09E-05	0.05	0.283	0.001	0.000705
HDL	rs9288418	rs3742599	2:211173358	14:64481661	0.377	0.173	0.018	0.00072	0.027	0.001564	-0.029	0.283	0.001	0.000709
HDL	rs10305724	rs344555	1:149061989	19:6630360	0.059	0.225	-0.008	0.495834	-0.013	0.020206	0.053	0.283	0.001	0.000751
HDL	rs9275312	rs1800766	6:32773706	3:149940332	0.138	0.171	0	0.953364	-0.017	0.014557	0.041	0.283	0.001	0.000754
HDL	rs11045310	rs17423306	12:20604966	11:103465519	0.462	0.175	0	0.988092	0.031	0.001294	-0.028	0.283	0.001	0.000767
HDL	rs1358753	rs6493059	6:161010560	15:40764818	0.137	0.216	0.026	0.001155	0.023	0.000153	-0.037	0.284	0.001	0.00077
HDL	rs4752805	rs3812265	11:47974931	7:134699344	0.25	0.243	-0.001	0.846345	-0.022	0.001015	0.028	0.284	0.001	0.000773
HDL	rs6663	rs12507396	12:108370986	4:156348494	0.251	0.117	0.006	0.295201	-0.028	0.001476	0.038	0.284	0.001	0.000776
HDL	rs253	rs3763959	8:19855697	17:22981461	0.451	0.421	0.027	9.15E-05	0.029	7.78E-05	-0.022	0.283	0.001	0.000782
HDL	rs8056954	rs1044250	16:55487730	19:8342164	0.178	0.314	-0.004	0.657957	0.022	9.49E-05	-0.03	0.284	0.001	0.000793
HDL	rs6586891	rs285	8:19958878	8:19859469	0.347	0.476	-0.003	0.643958	0.03	2.17E-06	-0.022	0.285	0.001	0.000794
HDL	rs180327	rs701106	11:116128869	12:123831589	0.371	0.168	-0.009	0.115364	0.023	0.008605	-0.029	0.284	0.001	0.000813
HDL	rs1063964	rs11606287	7:87673405	11:47364015	0.314	0.358	0.012	0.074614	0.011	0.09715	-0.023	0.284	0.001	0.000821
HDL	rs12454712	rs1342018	18:58996864	9:74971951	0.38	0.451	0.022	0.003104	0.023	0.000826	-0.022	0.283	0.001	0.000827
HDL	rs10501321	rs3960965	11:47251202	19:58481195	0.311	0.262	-0.005	0.431173	-0.021	0.003415	0.026	0.283	0.001	0.000833
HDL	rs12708967	rs5883	16:55550712	16:55564854	0.182	0.06	-0.072	4.82E-30	0.023	0.076296	0.048	0.291	0.001	0.000838
HDL	rs2239181	rs4796751	12:46542216	17:37381063	0.106	0.149	-0.025	0.002761	-0.018	0.009592	0.048	0.283	0.001	0.000847
HDL	rs10438303	rs4803766	15:41803709	19:50063008	0.099	0.43	0.013	0.277266	0.012	0.012689	-0.036	0.284	0.001	0.00085
HDL	rs230541	rs2274393	4:103716823	6:6127604	0.399	0.222	0.014	0.01327	0.023	0.004654	-0.026	0.283	0.001	0.000852
HDL	rs1514626	rs4765180	8:132834923	12:123884725	0.144	0.447	0.043	2.97E-05	0.019	0.000208	-0.03	0.284	0.001	0.000854
HDL	rs247615	rs158477	16:55542264	16:55565111	0.222	0.473	0.004	0.693608	0.031	3.28E-08	-0.025	0.285	0.001	0.000855
HDL	rs11902417	rs9295131	2:21052397	6:161056284	0.235	0.305	0.039	5.45E-08	-0.002	0.699498	-0.026	0.285	0.001	0.00087
HDL	rs3203922	rs2965101	5:118756852	19:49929652	0.28	0.323	0.021	0.002783	0.02	0.001294	-0.025	0.283	0.001	0.000892
HDL	rs2066716	rs11231745	9:106608526	11:63820235	0.088	0.141	0.033	0.000271	0.002	0.761344	-0.052	0.283	0.001	0.000893
HDL	rs1540037	rs255052	18:45436662	16:66582496	0.214	0.147	-0.013	0.036515	0.046	3.09E-09	-0.036	0.286	0.001	0.000898
HDL	rs4418728	rs6705820	10:94829714	2:69827241	0.461	0.486	-0.021	0.0065	-0.014	0.052853	0.021	0.283	0.001	0.000928
HDL	rs17410962	rs4795369	8:19892360	17:34862646	0.125	0.282	0.071	1.74E-15	-0.002	0.785298	-0.035	0.286	0.001	0.000966
HDL	rs4795369	rs2857078	17:34862646	17:39685697	0.282	0.309	-0.025	0.000169	-0.009	0.172483	0.025	0.283	0.001	0.000977
HDL	rs285	rs1131877	8:19859469	14:102411802	0.476	0.237	0.033	9.81E-09	0.017	0.057541	-0.025	0.284	0.001	0.000979
HDL	rs17410962	rs7459730	8:19892360	8:9034170	0.125	0.155	0.065	1.06E-16	-0.002	0.765662	-0.043	0.287	0.001	0.000982
HDL	rs10120087	rs10895596	9:106700971	11:103537253	0.11	0.302	0.037	9.85E-05	0.016	0.003515	-0.036	0.284	0.001	0.000985
HDL	rs34003087	rs2241201	11:116168806	12:108476515	0.057	0.274	0.066	2.69E-07	0.022	2.22E-05	-0.05	0.285	0.001	0.000991
HDL	rs263	rs865716	8:19857092	12:123856809	0.179	0.495	0.01	0.30171	-0.002	0.717734	0.027	0.285	0.001	0.000994
HDL	rs12720917	rs482548	16:55576893	11:61389758	0.162	0.094	0.004	0.587081	0.004	0.644025	0.048	0.284	0.001	0.000994
LDL	rs157580	rs405509	19:50087106	19:50100676	0.39	0.486	-0.27	1.14E-26	0.108	8.80E-09	0.088	0.069	0.002	3.93E-07
LDL	rs519113	rs405509	19:50068124	19:50100676	0.236	0.486	-0.168	5.19E-13	0.022	0.135921	0.098	0.062	0.002	5.29E-07
LDL	rs387976	rs405509	19:50070900	19:50100676	0.332	0.486	-0.174	1.02E-14	0.028	0.088092	0.086	0.063	0.002	1.24E-06
LDL	rs693	rs661665	2:21085700	2:21118646	0.5	0.492	-0.038	0.065004	0.056	0.006332	-0.081	0.064	0.002	1.33E-06
LDL	rs8102912	rs2738464	19:11066975	19:11103307	0.223	0.122	-0.126	1.37E-12	-0.122	2.55E-05	0.134	0.059	0.002	1.89E-06
LDL	rs1800479	rs661665	2:21080888	2:21118646	0.185	0.492	0.012	0.624385	0.038	0.007346	-0.101	0.059	0.002	2.51E-06

S1 Table, cont'd

Lipid	V1	V2	V1|chr:bp	V2|chr:bp	V1|MAF	V2|MAF	V1|Beta	V1|pval	V2|Beta	V2|pval	Int|Beta	Full|Rsq	Diff|Rsq	lrt|pval
LDL	rs1531517	rs519113	19:49934013	19:50068124	0.066	0.236	-0.001	0.96958	-0.035	0.01981	-0.165	0.061	0.001	7.98E-06
LDL	rs1531517	rs439401	19:49934013	19:50106291	0.066	0.371	-0.247	3.31E-14	-0.045	0.00049	0.159	0.059	0.001	8.70E-06
LDL	rs2518136	rs17069902	3:187820521	18:58182579	0.485	0.067	-0.008	0.53795	-0.156	0.00011	0.145	0.056	0.001	1.38E-05
LDL	rs1529729	rs5930	19:11024562	19:11085265	0.464	0.395	0.082	3.92E-06	0.022	0.2471	-0.073	0.058	0.001	1.82E-05
LDL	rs157580	rs439401	19:50087106	19:50106291	0.39	0.371	-0.138	2.16E-11	-0.041	0.05717	0.074	0.059	0.001	2.39E-05
LDL	rs2523451	rs630014	6:31477130	9:135139543	0.292	0.47	0.021	0.35975	0.010	0.51715	-0.077	0.057	0.001	2.50E-05
LDL	rs2273344	rs11206510	1:11027709	1:55268627	0.199	0.19	0.067	0.00018	0.000	0.98507	-0.112	0.057	0.001	2.72E-05
LDL	rs519113	rs439401	19:50068124	19:50106291	0.236	0.371	-0.148	6.66E-11	-0.046	0.00403	0.083	0.058	0.001	2.87E-05
LDL	rs17435152	rs3764261	7:40568630	16:55550825	0.149	0.32	-0.034	0.15002	-0.055	0.0002	0.103	0.057	0.001	4.56E-05
LDL	rs10179897	rs2965174	2:60958339	19:49936855	0.238	0.477	0.077	0.0011	0.003	0.83181	-0.080	0.057	0.001	5.74E-05
LDL	rs1531517	rs387976	19:49934013	19:50070900	0.066	0.332	-0.010	0.80378	-0.039	0.00368	-0.141	0.061	0.001	5.97E-05
LDL	rs496911	rs164632	12:121782422	19:4090849	0.403	0.266	0.023	0.14499	0.025	0.20835	-0.077	0.057	0.001	6.83E-05
LDL	rs3794991	rs7252981	19:19471596	19:19553579	0.086	0.337	0.088	0.10263	0.029	0.04316	-0.151	0.058	0.001	6.84E-05
LDL	rs10455872	rs678714	6:160930108	13:27964057	0.063	0.112	0.177	9.33E-11	0.026	0.18528	-0.203	0.058	0.001	6.95E-05
LDL	rs4965843	rs1946612	15:99750749	19:46459827	0.14	0.265	0.083	0.0002	0.045	0.00343	-0.107	0.056	0.001	8.00E-05
LDL	rs7448024	rs668842	5:63300839	15:46679257	0.1	0.306	-0.063	0.02045	0.010	0.47507	0.120	0.057	0.001	8.13E-05
LDL	rs5930	rs5742911	19:11085265	19:11104445	0.395	0.313	-0.089	4.37E-07	-0.088	4.21E-05	0.071	0.058	0.001	8.85E-05
LDL	rs6859355	rs11551870	5:52337473	14:31695603	0.432	0.097	0.011	0.40448	-0.051	0.1135	0.111	0.057	0.001	9.58E-05
LDL	rs873308	rs4380146	1:25631242	19:51798474	0.459	0.311	0.010	0.52822	0.074	0.00031	-0.070	0.057	0.001	9.69E-05
LDL	rs17149647	rs10808071	7:86924867	7:86978744	0.142	0.194	-0.100	1.70E-05	-0.046	0.01452	0.106	0.056	0.001	0.00013
LDL	rs12464355	rs13094915	2:118566320	3:52482759	0.096	0.393	0.053	0.07624	0.043	0.00122	-0.111	0.056	0.001	0.00013
LDL	rs9275312	rs13465	6:32773706	19:10663792	0.138	0.06	-0.069	0.00024	-0.136	1.49E-06	0.190	0.057	0.001	0.00013
LDL	rs9469220	rs11085258	6:32766288	19:19150830	0.495	0.383	-0.030	0.10383	-0.072	0.00048	0.064	0.056	0.001	0.00013
LDL	rs12410114	rs12915677	1:235837714	15:46652089	0.284	0.198	0.011	0.48659	0.063	0.0016	-0.089	0.056	0.001	0.00015
LDL	rs6657811	rs1531517	1:109608806	19:49934013	0.129	0.066	-0.102	6.72E-08	-0.103	0.00011	-0.195	0.063	0.001	0.00016
LDL	rs1167998	rs860867	1:62704220	12:42961663	0.336	0.11	-0.017	0.2096	0.036	0.17188	-0.106	0.057	0.001	0.00017
LDL	rs17035630	rs27184	1:109612504	5:58611080	0.121	0.447	0.138	3.26E-06	0.035	0.00879	-0.097	0.056	0.001	0.00019
LDL	rs12410114	rs3796529	1:235837714	4:57492171	0.284	0.188	0.011	0.51678	0.039	0.05547	-0.087	0.056	0.001	0.00019
LDL	rs2479413	rs27183	1:55291270	5:58624386	0.348	0.433	-0.002	0.92917	0.035	0.04295	-0.066	0.058	0.001	0.0002
LDL	rs187645	rs12708974	5:58647274	16:55563051	0.08	0.113	0.036	0.14036	0.079	0.0001	-0.176	0.056	0.001	0.00021
LDL	rs949425	rs10774613	7:141724336	12:110030548	0.107	0.44	-0.051	0.09215	-0.019	0.15138	0.100	0.056	0.001	0.00021
LDL	rs17035443	rs611917	1:109590236	1:109616775	0.196	0.32	0.035	0.09894	-0.084	7.37E-08	-0.082	0.062	0.001	0.00022
LDL	rs2066717	rs11040883	9:106631299	11:6366366	0.062	0.151	0.024	0.3946	-0.029	0.09679	0.172	0.057	0.001	0.00023
LDL	rs6531594	rs3851615	4:37570935	12:69812331	0.053	0.449	0.084	0.05189	0.043	0.00061	-0.138	0.057	0.001	0.00023
LDL	rs4630309	rs7968824	11:66089648	12:42849702	0.242	0.053	-0.029	0.04024	-0.082	0.01364	0.161	0.056	0.001	0.00024
LDL	rs916055	rs8102530	17:4481583	19:19671078	0.33	0.419	0.036	0.06056	0.014	0.40051	-0.065	0.057	0.001	0.00024
LDL	rs17035443	rs7515901	1:109590236	1:109641419	0.196	0.164	0.013	0.4694	-0.001	0.95346	-0.097	0.057	0.001	0.00025
LDL	rs4299376	rs11085098	2:43926080	19:4735553	0.312	0.342	0.135	1.05E-13	0.025	0.14543	-0.069	0.060	0.001	0.00025
LDL	rs3794991	rs2426515	19:19471596	20:52506124	0.086	0.214	-0.041	0.11688	0.050	0.00142	-0.135	0.058	0.001	0.00026
LDL	rs10402271	rs2075650	19:50021054	19:50087459	0.331	0.144	0.073	9.65E-07	0.200	2.66E-13	-0.089	0.062	0.001	0.00026
LDL	rs3761739	rs3828699	5:74667257	5:126189165	0.148	0.206	0.000	0.98545	-0.067	6.98E-05	0.107	0.057	0.001	0.00026
LDL	rs6413504	rs5742911	19:11102915	19:11104445	0.473	0.313	0.069	2.77E-05	0.027	0.17351	-0.070	0.056	0.001	0.00027
LDL	rs624612	rs629001	1:55290471	1:109640441	0.419	0.068	0.031	0.01749	0.020	0.58391	-0.121	0.057	0.001	0.00028
LDL	rs1994450	rs10144857	11:103302559	14:34830835	0.165	0.162	-0.036	0.0597	-0.008	0.67729	0.113	0.056	0.001	0.00028
LDL	rs11784581	rs2149860	8:141736403	13:32526989	0.496	0.447	0.082	2.28E-05	0.066	0.00124	-0.061	0.057	0.001	0.00028
LDL	rs499883	rs7646621	1:55291762	3:185123559	0.379	0.48	-0.141	9.20E-12	-0.044	0.01616	0.062	0.059	0.001	0.00029
LDL	rs10744777	rs6602910	12:110717401	13:113564928	0.322	0.381	-0.049	0.00934	-0.040	0.01774	0.067	0.056	0.001	0.0003
LDL	rs10402271	rs405509	19:50021054	19:50100676	0.331	0.486	0.134	1.71E-10	0.119	9.37E-13	-0.064	0.061	0.001	0.0003
LDL	rs2149860	rs36232	13:32526989	16:2139789	0.447	0.186	0.034	0.0175	0.104	1.64E-05	-0.077	0.056	0.001	0.00031
LDL	rs10837094	rs174570	11:4972062	11:61353788	0.331	0.132	-0.047	0.00111	-0.108	1.57E-05	0.094	0.056	0.001	0.00031
LDL	rs2372565	rs916055	2:216066837	17:4481583	0.254	0.33	-0.050	0.00814	-0.054	0.00079	0.073	0.056	0.001	0.00031
LDL	rs10509954	rs4803789	10:113658378	19:50215209	0.091	0.351	0.092	0.00218	0.005	0.72197	-0.110	0.055	0.001	0.00032
LDL	rs12410114	rs3171425	1:235837714	21:33590617	0.284	0.411	0.032	0.11774	0.068	2.17E-05	-0.068	0.057	0.001	0.00032
LDL	rs2228603	rs5924611	19:19190924	23:16801918	0.077	0.422	-0.167	1.33E-07	0.009	0.38235	0.096	0.057	0.001	0.00032

S1 Table, cont'd

Lipid	V1	V2	V1|chr:bp	V2|chr:bp	V1|MAF	V2|MAF	V1|Beta	V1|pval	V2|Beta	V2|pval	Int|Beta	Full|Rsq	Diff|Rsq	lrt|pval
LDL	rs1531517	rs405509	19:49934013	19:50100676	0.066	0.486	-0.233	1.28E-10	0.048	0.00013	0.123	0.061	0.001	0.00033
LDL	rs949425	rs676160	7:141724336	11:120845443	0.107	0.089	0.066	0.00167	0.011	0.63651	-0.173	0.056	0.001	0.00033
LDL	rs2495498	rs10402271	1:55267951	19:50021054	0.114	0.331	0.108	3.90E-05	0.088	3.82E-10	-0.100	0.058	0.001	0.00034
LDL	rs728614	rs231914	1:227630740	11:2705952	0.193	0.396	0.099	1.73E-05	0.018	0.21701	-0.078	0.056	0.001	0.00034
LDL	rs519113	rs2889490	19:50068124	19:50242247	0.236	0.49	-0.010	0.68174	0.019	0.21289	-0.070	0.058	0.001	0.00035
LDL	rs6414624	rs821959	4:5794413	10:108444367	0.2	0.062	-0.030	0.05661	-0.077	0.01175	0.155	0.056	0.001	0.00037
LDL	rs1800479	rs36232	2:21080888	16:2139789	0.185	0.186	-0.119	8.64E-11	0.001	0.95563	0.098	0.058	0.001	0.00037
LDL	rs1055259	rs2738464	1:110078384	19:11103307	0.123	0.122	0.006	0.77489	-0.028	0.17619	-0.139	0.056	0.001	0.00037
LDL	rs12467409	rs27184	2:203102593	5:58611080	0.115	0.447	-0.060	0.04798	-0.011	0.42612	0.096	0.056	0.001	0.00037
LDL	rs4803766	rs157580	19:50063008	19:50087106	0.431	0.39	0.016	0.35856	-0.016	0.38689	-0.061	0.059	0.001	0.00037
LDL	rs3212576	rs6947240	5:52402662	7:150288142	0.184	0.238	0.080	4.57E-05	0.053	0.0013	-0.089	0.057	0.001	0.00038
LDL	rs873308	rs6933	1:25631242	17:61638692	0.459	0.452	0.020	0.29337	0.065	0.00084	-0.059	0.057	0.001	0.00038
LDL	rs7552841	rs8184236	1:55291340	20:2987115	0.383	0.472	-0.001	0.94374	-0.060	0.00084	0.061	0.058	0.001	0.0004
LDL	rs4299376	rs860867	2:43926080	12:42961663	0.312	0.11	0.109	1.45E-14	0.031	0.23508	-0.103	0.060	0.001	0.0004
LDL	rs1804506	rs4969170	1:91920601	17:73872133	0.145	0.342	0.069	0.00397	0.022	0.12066	-0.088	0.056	0.001	0.00041
LDL	rs2238675	rs7252981	19:19197608	19:19553579	0.134	0.337	0.048	0.08654	0.016	0.26194	-0.089	0.056	0.001	0.00041
LDL	rs13376356	rs12039600	1:2396747	1:160426106	0.116	0.122	0.005	0.79627	0.000	0.99118	-0.138	0.056	0.001	0.00042
LDL	rs6859355	rs1483299	5:52337473	15:93697160	0.432	0.24	0.000	0.99088	-0.031	0.1596	0.070	0.057	0.001	0.00043
LDL	rs7709325	rs231914	5:78350811	11:2705952	0.184	0.396	-0.047	0.0388	-0.040	0.00641	0.077	0.056	0.001	0.00044
LDL	rs10028670	rs13465	4:142848174	19:10663792	0.27	0.06	-0.001	0.95266	-0.006	0.86025	-0.142	0.057	0.001	0.00044
LDL	rs11206514	rs12811752	1:55288592	12:20469072	0.391	0.419	0.023	0.20872	0.085	2.40E-06	-0.060	0.057	0.001	0.00046
LDL	rs13182800	rs916055	5:142781673	17:4481583	0.202	0.33	0.023	0.26584	0.013	0.39233	-0.076	0.056	0.001	0.00046
LDL	rs629001	rs10882273	1:109640441	10:95338895	0.068	0.389	0.011	0.76236	0.009	0.46846	-0.117	0.057	0.001	0.00046
LDL	rs6453133	rs16948381	5:74728532	17:45235426	0.302	0.063	0.077	1.42E-08	0.131	8.68E-05	-0.127	0.058	0.001	0.00048
LDL	rs2228603	rs5969730	19:19190924	23:16783571	0.077	0.402	-0.159	1.63E-07	0.014	0.17924	0.092	0.058	0.001	0.00048
LDL	rs4299376	rs290485	2:43926080	10:114899910	0.312	0.173	0.116	2.75E-14	0.050	0.02087	-0.083	0.059	0.001	0.00049
LDL	rs9640378	rs10837094	7:142775040	11:4972062	0.228	0.331	0.035	0.07819	0.011	0.49656	-0.076	0.056	0.001	0.0005
LDL	rs10744777	rs8102912	12:110717401	19:11066975	0.322	0.223	-0.031	0.04861	-0.136	8.46E-12	0.074	0.059	0.001	0.0005
LDL	rs892023	rs857240	19:19524850	20:3023629	0.399	0.099	-0.026	0.05166	-0.036	0.2265	0.098	0.056	0.001	0.00051
LDL	rs11206514	rs6413504	1:55288592	19:11102915	0.391	0.473	-0.083	5.65E-05	-0.005	0.78418	0.060	0.056	0.001	0.00052
LDL	rs3793342	rs4082919	7:150326128	17:73889077	0.151	0.489	-0.070	0.01535	-0.039	0.00515	0.081	0.055	0.001	0.00052
LDL	rs11706810	rs678714	3:161642615	13:27964057	0.483	0.112	0.003	0.81166	0.087	0.00597	-0.091	0.056	0.001	0.00052
LDL	rs6428291	rs2738456	1:187592960	19:11097804	0.05	0.33	0.125	0.00131	-0.006	0.64004	-0.140	0.056	0.001	0.00053
LDL	rs3087465	rs11954894	3:30622164	5:50709888	0.223	0.257	-0.054	0.00303	-0.024	0.16342	0.079	0.056	0.001	0.00054
LDL	rs4808199	rs5969730	19:19406099	23:16783571	0.178	0.402	-0.081	0.00016	0.007	0.53464	0.063	0.057	0.001	0.00054
LDL	rs6970522	rs12708974	7:151080486	16:55563051	0.486	0.113	-0.011	0.41556	-0.038	0.23022	0.090	0.056	0.001	0.00056
LDL	rs2516498	rs7968824	6:31573878	12:42849702	0.144	0.053	-0.001	0.94393	-0.061	0.04781	0.186	0.056	0.001	0.00056
LDL	rs11752651	rs1529729	6:41668265	19:11024562	0.056	0.464	0.163	6.85E-05	0.052	2.78E-05	-0.121	0.057	0.001	0.00057
LDL	rs4307284	rs3807370	7:81324694	7:150304247	0.16	0.362	0.085	0.00028	0.052	0.00037	-0.081	0.056	0.001	0.00057
LDL	rs10888896	rs2738464	1:55281801	19:11103307	0.253	0.122	-0.013	0.4027	-0.009	0.70645	-0.101	0.056	0.001	0.00059
LDL	rs4808199	rs7252981	19:19406099	19:19553579	0.178	0.337	0.049	0.07831	0.024	0.12803	-0.078	0.056	0.001	0.0006
LDL	rs4728737	rs9747201	7:87677464	17:77771141	0.053	0.315	-0.074	0.04649	-0.031	0.01961	0.136	0.056	0.001	0.0006
LDL	rs3828699	rs3184504	5:126189165	12:110368991	0.206	0.493	-0.105	2.45E-05	-0.050	0.00047	0.070	0.057	0.001	0.0006
LDL	rs907604	rs2803899	11:1805930	14:37743966	0.377	0.068	-0.045	0.0005	-0.096	0.00482	0.118	0.056	0.001	0.00061
LDL	rs754523	rs12467409	2:21165196	2:203102593	0.319	0.115	0.079	2.87E-08	-0.032	0.20773	0.095	0.061	0.001	0.00062
LDL	rs17035630	rs27183	1:109612504	5:58624386	0.121	0.433	-0.028	0.32366	-0.033	0.01376	0.087	0.057	0.001	0.00064
LDL	rs10127790	rs2071429	1:109692656	23:153413702	0.293	0.132	-0.029	0.04365	0.051	0.0087	-0.078	0.056	0.001	0.00064
LDL	rs2516498	rs860867	6:31573878	12:42961663	0.144	0.11	-0.011	0.57522	-0.072	0.00103	0.129	0.056	0.001	0.00065
LDL	rs2273344	rs37602	1:11027709	16:74062547	0.199	0.142	-0.004	0.80785	-0.062	0.00268	0.103	0.056	0.001	0.00065
LDL	rs8102912	rs1433099	19:11066975	19:11103658	0.223	0.268	-0.131	3.32E-11	-0.065	0.00017	0.075	0.059	0.001	0.00066
LDL	rs3828699	rs2965101	5:126189165	19:49929652	0.206	0.323	-0.083	3.57E-05	-0.048	0.00208	0.074	0.056	0.001	0.00066
LDL	rs17035443	rs5930	1:109590236	19:11085265	0.196	0.395	-0.091	7.04E-05	-0.077	1.91E-07	0.074	0.057	0.001	0.00068
LDL	rs2301128	rs5110	11:113673209	11:116196844	0.195	0.082	-0.041	0.01097	-0.097	0.00021	0.126	0.056	0.001	0.00069
LDL	rs10127790	rs668842	1:109692656	15:46679257	0.293	0.306	-0.094	2.03E-07	-0.005	0.76888	0.067	0.057	0.001	0.00072

S1 Table, cont'd

Lipid	V1	V2	V1|chr:bp	V2|chr:bp	V1|MAF	V2|MAF	V1|Beta	V1|pval	V2|Beta	V2|pval	Int|Beta	Full|Rsq	Diff|Rsq	lrt|pval
LDL	rs624249	rs12708974	6:160599390	16:55563051	0.414	0.113	-0.041	0.00243	-0.028	0.34457	0.090	0.056	0.001	0.00073
LDL	rs842207	rs8184236	12:42751537	20:2987115	0.144	0.472	0.043	0.12531	0.012	0.39808	-0.080	0.056	0.001	0.00073
LDL	rs1410997	rs6494006	1:194943786	15:56517863	0.422	0.063	0.032	0.01152	0.128	0.00092	-0.117	0.056	0.001	0.00075
LDL	rs4728737	rs9900564	7:87677464	17:73889053	0.053	0.348	0.105	0.0051	-0.006	0.6573	-0.129	0.056	0.001	0.00076
LDL	rs2523451	rs657152	6:31477130	9:135129086	0.292	0.371	-0.098	2.63E-06	0.006	0.69798	0.063	0.057	0.001	0.00076
LDL	rs1738475	rs3087465	1:23409478	3:30622164	0.418	0.223	-0.005	0.74003	-0.070	0.0014	0.068	0.056	0.001	0.00076
LDL	rs2257096	rs4964731	1:235770024	12:107581938	0.415	0.479	-0.089	9.05E-06	-0.059	0.00121	0.057	0.057	0.001	0.00078
LDL	rs709149	rs4965843	3:12425354	15:99750749	0.383	0.14	-0.033	0.01921	-0.038	0.13332	0.083	0.056	0.001	0.00078
LDL	rs4803766	rs2889490	19:50063008	19:50242247	0.431	0.49	0.032	0.11775	0.037	0.04786	-0.057	0.056	0.001	0.00079
LDL	rs4299376	rs4806998	2:43926080	19:4747782	0.312	0.464	0.143	7.55E-12	0.025	0.12247	-0.060	0.059	0.001	0.00079
LDL	rs661665	rs2066717	2:21118646	9:106631299	0.492	0.062	-0.010	0.43124	-0.031	0.44964	0.112	0.057	0.001	0.00081
LDL	rs821959	rs37602	10:108444367	16:74062547	0.062	0.142	-0.060	0.03651	-0.042	0.0192	0.165	0.056	0.001	0.00082
LDL	rs12136600	rs3129875	1:55293783	6:32515446	0.072	0.289	-0.145	1.49E-06	0.021	0.1898	0.119	0.057	0.001	0.00083
LDL	rs4969170	rs1054564	17:73872133	19:18360815	0.342	0.143	-0.027	0.05809	-0.094	8.92E-05	0.084	0.056	0.001	0.00083
LDL	rs3753658	rs7481967	1:224079309	11:1289978	0.129	0.16	-0.055	0.00823	-0.039	0.03347	0.115	0.056	0.001	0.00083
LDL	rs4673999	rs8176720	2:216058272	9:135122694	0.445	0.335	-0.039	0.02067	-0.075	0.00019	0.059	0.056	0.001	0.00084
LDL	rs7546522	rs2238086	1:55289301	12:2482502	0.157	0.092	-0.029	0.09889	-0.021	0.36286	0.135	0.056	0.001	0.00084
LDL	rs9469220	rs1483299	6:32766288	15:93697160	0.495	0.24	-0.013	0.43338	-0.034	0.14838	0.064	0.056	0.001	0.00086
LDL	rs9273363	rs5110	6:32734250	11:116196844	0.269	0.082	-0.013	0.43053	0.016	0.57612	-0.112	0.056	0.001	0.00086
LDL	rs939335	rs6859	3:185228357	19:50073874	0.491	0.419	-0.038	0.04191	0.014	0.503	0.057	0.058	0.001	0.00087
LDL	rs480780	rs422674	13:32505319	22:22736778	0.388	0.36	0.034	0.05387	0.031	0.09635	-0.059	0.056	0.001	0.00087
LDL	rs10208987	rs164632	2:43896639	19:4090849	0.082	0.266	-0.112	8.76E-05	-0.054	0.00018	0.117	0.057	0.001	0.00087
LDL	rs4896044	rs4808199	6:134779781	19:19406099	0.414	0.178	-0.022	0.12089	-0.092	0.00016	0.074	0.056	0.001	0.00087
LDL	rs2001945	rs8102530	8:126547160	19:19671078	0.481	0.419	0.097	2.40E-07	0.025	0.21778	-0.056	0.057	0.001	0.00088
LDL	rs12039600	rs9640378	1:160426106	7:142775040	0.122	0.228	-0.079	0.00056	-0.037	0.01917	0.103	0.056	0.001	0.00088
LDL	rs6428291	rs206079	1:187592960	13:31818618	0.05	0.46	-0.099	0.03179	0.013	0.30287	0.129	0.056	0.001	0.00088
LDL	rs2228603	rs3794991	19:19190924	19:19471596	0.077	0.086	0.070	0.10271	-0.017	0.65479	-0.154	0.058	0.001	0.00089
LDL	rs422674	rs2318792	22:22736778	23:24002625	0.36	0.316	-0.048	0.00224	-0.060	8.70E-05	0.051	0.056	0.001	0.00089
LDL	rs10888896	rs6413504	1:55281801	19:11102915	0.253	0.473	-0.101	1.57E-05	0.009	0.55356	0.065	0.055	0.001	0.00089
LDL	rs2228671	rs11085258	19:11071912	19:19150830	0.119	0.383	-0.205	1.14E-13	-0.029	0.03377	0.087	0.060	0.001	0.0009
LDL	rs4808199	rs5924611	19:19406099	23:16801918	0.178	0.422	-0.081	0.00025	0.002	0.83468	0.060	0.056	0.001	0.0009
LDL	rs3212576	rs1483299	5:52402662	15:93697160	0.184	0.24	-0.003	0.88067	-0.002	0.90452	0.085	0.057	0.001	0.00093
LDL	rs17435152	rs8102912	7:40568630	19:11066975	0.149	0.223	-0.009	0.66006	-0.116	1.76E-12	0.094	0.059	0.001	0.00093
LDL	rs10888896	rs2066716	1:55281801	9:106608526	0.253	0.088	-0.018	0.23587	0.108	6.81E-05	-0.110	0.056	0.001	0.00093
LDL	rs11206510	rs2738459	1:55268627	19:11099473	0.19	0.475	0.024	0.34162	0.008	0.57578	-0.071	0.057	0.001	0.00095
LDL	rs27184	rs12708974	5:58611080	16:55563051	0.447	0.113	-0.007	0.59612	-0.032	0.29666	0.088	0.056	0.001	0.00096
LDL	rs4673993	rs2318792	2:215920584	23:24002625	0.328	0.316	0.033	0.04266	0.012	0.43399	-0.053	0.056	0.001	0.00096
LDL	rs17398765	rs4942443	2:21124256	13:31817526	0.07	0.19	0.132	2.70E-06	-0.028	0.08101	-0.138	0.057	0.001	0.00096
LDL	rs7265992	rs946252	20:32989068	23:11222948	0.161	0.317	-0.017	0.39277	-0.038	0.0021	0.067	0.055	0.001	0.00096
LDL	rs949425	rs231914	7:141724336	11:2705952	0.107	0.396	0.107	0.00022	0.008	0.57451	-0.090	0.056	0.001	0.00099
LDL	rs630431	rs1697845	1:55299911	5:75958260	0.302	0.343	0.005	0.79917	0.062	0.0003	-0.064	0.057	0.001	0.00099
LDL	rs2980885	rs4898398	8:126543488	23:153732140	0.23	0.092	0.009	0.56145	-0.056	0.01035	0.092	0.056	0.001	0.00099
LDL	rs36232	rs1054564	16:2139789	19:18360815	0.186	0.143	0.064	0.00021	0.001	0.96405	-0.101	0.056	0.001	0.00099
LDL	rs2779360	rs520354	1:235757546	2:21113117	0.481	0.47	0.072	0.00019	-0.016	0.42057	-0.054	0.058	0.001	0.00099
LDL	rs4673993	rs2965101	2:215920584	19:49929652	0.328	0.323	-0.042	0.01796	-0.058	0.00101	0.064	0.056	0.001	0.001
LDL	rs17496549	rs2228603	6:32517686	19:19190924	0.112	0.077	0.054	0.00893	-0.050	0.04623	-0.160	0.057	0.001	0.001
TC	rs8102912	rs2738464	19:11066975	19:11103307	0.223	0.122	-0.109	1.08E-08	-0.139	8.64E-06	0.135	0.084	0.001	8.85E-06
TC	rs180327	rs10750097	11:116128869	11:116169250	0.37	0.208	0.010	0.54004	-0.075	0.00458	0.100	0.085	0.001	9.45E-06
TC	rs4938303	rs180327	11:116090197	11:116128869	0.271	0.37	-0.049	0.03107	0.001	0.94063	0.091	0.084	0.001	1.02E-05
TC	rs498793	rs2331291	11:61381281	22:25388821	0.401	0.137	-0.002	0.90197	-0.105	0.00016	0.113	0.085	0.001	1.63E-05
TC	rs1531517	rs519113	19:49934013	19:50068124	0.067	0.238	0.043	0.28494	-0.032	0.05307	-0.171	0.086	0.001	1.80E-05
TC	rs28927680	rs387976	11:116124283	19:50070900	0.069	0.334	-0.047	0.18666	-0.084	8.20E-09	0.159	0.085	0.001	1.81E-05
TC	rs6892794	rs12939509	5:149104324	17:64960981	0.256	0.262	-0.041	0.03025	-0.042	0.02408	0.098	0.084	0.001	1.94E-05
TC	rs10888896	rs2066716	1:55281801	9:106608526	0.252	0.09	-0.013	0.41858	0.138	2.51E-06	-0.153	0.084	0.001	2.02E-05

S1 Table, cont'd

Lipid	V1	V2	V1|chr:bp	V2|chr:bp	V1|MAF	V2|MAF	V1|Beta	V1|pval	V2|Beta	V2|pval	Int|Beta	Full|Rsq	Diff|Rsq	lrt|pval
TC	rs664971	rs2331291	11:116576067	22:25388821	0.243	0.137	-0.004	0.83316	-0.075	0.00115	0.127	0.084	0.001	2.09E-05
TC	rs2072183	rs1859223	7:44545705	17:39435091	0.228	0.158	-0.012	0.51152	-0.047	0.03195	0.120	0.084	0.001	3.27E-05
TC	rs481843	rs12820008	11:116031077	12:101676159	0.082	0.285	0.146	2.27E-06	-0.005	0.73904	-0.149	0.084	0.001	3.87E-05
TC	rs9469220	rs11085258	6:32766288	19:19150830	0.499	0.382	-0.031	0.11839	-0.082	0.00025	0.074	0.084	0.001	4.28E-05
TC	rs2952975	rs2096362	17:26515118	23:153885468	0.155	0.26	-0.072	0.00084	-0.035	0.01149	0.098	0.083	0.001	4.30E-05
TC	rs2238675	rs7252981	19:19197608	19:19553579	0.132	0.338	0.041	0.17563	0.032	0.04369	-0.110	0.084	0.001	4.95E-05
TC	rs873308	rs4380146	1:25631242	19:51798474	0.463	0.309	0.014	0.42446	0.074	0.00091	-0.078	0.084	0.001	4.96E-05
TC	rs4705411	rs4148809	5:149411218	7:86941199	0.154	0.459	-0.070	0.01645	-0.048	0.00123	0.103	0.084	0.001	5.13E-05
TC	rs17435152	rs3764261	7:40568630	16:55550825	0.147	0.322	-0.034	0.17736	0.022	0.17185	0.110	0.085	0.001	5.32E-05
TC	rs4664446	rs12501467	2:162618649	4:5794880	0.484	0.135	-0.056	0.00013	-0.117	0.00015	0.104	0.084	0.001	6.36E-05
TC	rs2238675	rs1469713	19:19197608	19:19389806	0.132	0.354	0.044	0.1622	0.032	0.04051	-0.108	0.084	0.001	6.75E-05
TC	rs3794991	rs7252981	19:19471596	19:19553579	0.085	0.338	0.060	0.29912	0.046	0.00259	-0.163	0.086	0.001	6.77E-05
TC	rs10750097	rs2149860	11:116169250	13:32526989	0.208	0.45	0.114	6.72E-06	0.033	0.03701	-0.088	0.084	0.001	6.87E-05
TC	rs180327	rs6589568	11:116128869	11:116175948	0.37	0.21	0.020	0.23494	-0.042	0.06762	0.089	0.085	0.001	8.35E-05
TC	rs717326	rs13465	9:21948524	19:10663792	0.094	0.061	-0.023	0.32244	-0.033	0.25675	-0.258	0.084	0.001	8.48E-05
TC	rs11752651	rs1529729	6:41668265	19:11024562	0.056	0.464	0.194	1.10E-05	0.055	4.69E-05	-0.148	0.084	0.001	9.06E-05
TC	rs657420	rs13182800	1:109627659	5:142781673	0.477	0.204	-0.023	0.14155	0.049	0.06494	-0.086	0.085	0.001	9.28E-05
TC	rs12136600	rs2296616	1:55293783	10:91342946	0.073	0.456	-0.185	2.47E-06	0.010	0.46429	0.137	0.084	0.001	9.61E-05
TC	rs6433688	rs4144739	2:178245922	8:32089597	0.141	0.498	-0.076	0.01892	-0.019	0.19839	0.102	0.084	0.001	0.0001
TC	rs2980885	rs4898398	8:126543488	23:153732140	0.233	0.092	0.016	0.31548	-0.082	0.00051	0.117	0.084	0.001	0.00011
TC	rs693	rs661665	2:21085700	2:21118646	0.496	0.493	0.041	0.06392	-0.090	6.33E-05	0.070	0.088	0.001	0.00011
TC	rs6657811	rs2228671	1:109608806	19:11071912	0.13	0.12	-0.090	2.48E-05	-0.075	0.00075	-0.161	0.089	0.001	0.00011
TC	rs13005416	rs3759324	2:215904290	12:6355922	0.466	0.24	-0.040	0.01459	-0.076	0.00207	0.081	0.083	0.001	0.00011
TC	rs898034	rs2241201	2:27944324	12:108476515	0.209	0.274	-0.093	5.45E-06	0.001	0.94896	0.094	0.084	0.001	0.00012
TC	rs11216129	rs10750097	11:116125466	11:116169250	0.124	0.208	0.035	0.24845	0.088	8.83E-06	-0.116	0.084	0.001	0.00013
TC	rs1800479	rs36232	2:21080888	16:2139789	0.184	0.19	-0.117	3.39E-09	-0.002	0.93356	0.114	0.085	0.001	0.00013
TC	rs11740792	rs11826287	5:142727513	11:67903237	0.166	0.183	-0.067	0.00138	-0.085	1.71E-05	0.121	0.084	0.001	0.00014
TC	rs3129875	rs2607336	6:32515446	19:60495632	0.285	0.493	0.120	2.30E-06	0.044	0.01068	-0.075	0.084	0.001	0.00016
TC	rs290485	rs2275998	10:114899910	11:66083157	0.175	0.179	-0.040	0.04978	-0.044	0.02721	0.118	0.081	0.001	0.00016
TC	rs1152002	rs2071437	3:12446871	12:46673186	0.485	0.216	0.022	0.16396	0.096	0.0003	-0.083	0.083	0.001	0.00017
TC	rs9804646	rs454715	11:116170289	19:53806048	0.08	0.399	0.047	0.19939	0.037	0.00912	-0.130	0.084	0.001	0.00017
TC	rs7306218	rs12721046	12:108532674	19:50113094	0.201	0.141	0.053	0.00383	0.192	3.44E-18	-0.118	0.087	0.001	0.00017
TC	rs519113	rs405509	19:50068124	19:50100676	0.238	0.482	-0.144	1.15E-08	0.004	0.80104	0.080	0.086	0.001	0.00017
TC	rs6450517	rs1529729	5:58686201	19:11024562	0.252	0.464	0.093	0.00011	0.075	3.40E-06	-0.077	0.084	0.001	0.00018
TC	rs7306218	rs10402271	12:108532674	19:50021054	0.201	0.331	0.078	0.00054	0.094	1.19E-08	-0.089	0.085	0.001	0.00018
TC	rs2066717	rs16924332	9:106631299	12:21865495	0.062	0.347	0.185	9.47E-07	0.022	0.12065	-0.148	0.084	0.001	0.00018
TC	rs3790508	rs11902417	1:149942110	2:21052397	0.128	0.238	0.056	0.02119	-0.021	0.23015	-0.118	0.084	0.001	0.00018
TC	rs9266831	rs675	6:31491062	11:116196885	0.264	0.195	-0.002	0.91168	-0.058	0.00604	0.095	0.083	0.001	0.00018
TC	rs231914	rs9892809	11:2705952	17:9815657	0.392	0.483	0.054	0.01435	0.040	0.03803	-0.068	0.083	0.001	0.0002
TC	rs17244848	rs9369425	5:74679426	6:43918952	0.099	0.291	0.125	1.10E-05	-0.009	0.55529	-0.124	0.084	0.001	0.0002
TC	rs11122458	rs6986495	1:228375478	8:26747282	0.218	0.183	0.001	0.93943	0.032	0.11649	-0.103	0.084	0.001	0.00021
TC	rs11254269	rs689243	10:16979846	11:116227903	0.09	0.367	-0.139	1.86E-05	0.028	0.05165	0.120	0.084	0.001	0.00022
TC	rs323881	rs2607336	3:51937121	19:60495632	0.059	0.493	0.136	0.00338	0.016	0.22058	-0.141	0.083	0.001	0.00023
TC	rs7306218	rs157580	12:108532674	19:50087106	0.201	0.392	-0.046	0.05047	-0.109	1.00E-11	0.084	0.086	0.001	0.00023
TC	rs6859355	rs2353082	5:52337473	7:72551104	0.43	0.272	-0.006	0.72658	-0.036	0.11959	0.075	0.084	0.001	0.00023
TC	rs10986783	rs4942443	9:127317563	13:31817526	0.056	0.189	0.095	0.00487	-0.020	0.2469	-0.179	0.083	0.001	0.00024
TC	rs6589602	rs2331291	11:116542275	22:25388821	0.395	0.137	0.013	0.3997	-0.091	0.00105	0.098	0.084	0.001	0.00024
TC	rs10799528	rs12939509	1:227628059	17:64960981	0.474	0.262	-0.001	0.95511	0.080	0.0009	-0.074	0.084	0.001	0.00025
TC	rs4938303	rs2149860	11:116090197	13:32526989	0.271	0.45	0.105	5.58E-06	0.036	0.03316	-0.074	0.082	0.001	0.00025
TC	rs13501	rs2385114	6:32901501	8:126516490	0.321	0.494	0.110	4.17E-06	0.075	2.29E-05	-0.070	0.084	0.001	0.00026
TC	rs2952975	rs1531517	17:26515118	19:49934013	0.155	0.067	0.003	0.85887	-0.049	0.10292	-0.177	0.085	0.001	0.00026
TC	rs17655652	rs2275998	7:44547516	11:66083157	0.316	0.179	0.018	0.25815	0.057	0.01432	-0.092	0.081	0.001	0.00027
TC	rs13465	rs11085258	19:10663792	19:19150830	0.061	0.382	-0.187	2.90E-06	-0.025	0.07044	0.142	0.084	0.001	0.00027
TC	rs2495498	rs10402271	1:55267951	19:50021054	0.117	0.331	0.119	2.75E-05	0.084	3.56E-08	-0.110	0.085	0.001	0.00027

S1 Table, cont'd

Lipid	V1	V2	V1|chr:bp	V2|chr:bp	V1|MAF	V2|MAF	V1|Beta	V1|pval	V2|Beta	V2|pval	Int|Beta	Full|Rsq	Diff|Rsq	lrt|pval
TC	rs1800479	rs661665	2:21080888	2:21118646	0.184	0.493	0.005	0.8611	0.035	0.02089	-0.085	0.085	0.001	0.00027
TC	rs13219571	rs2075290	6:132320836	11:116158506	0.228	0.072	0.010	0.53071	0.173	1.71E-08	-0.152	0.085	0.001	0.00029
TC	rs520354	rs594418	2:21113117	10:84375032	0.467	0.186	-0.096	3.85E-10	-0.105	0.00011	0.083	0.085	0.001	0.00029
TC	rs6657811	rs1531517	1:109608806	19:49934013	0.13	0.067	-0.103	4.11E-07	-0.060	0.03704	-0.203	0.088	0.001	0.00029
TC	rs10208987	rs1540037	2:43896639	18:45436662	0.083	0.213	-0.116	7.37E-05	-0.059	0.00044	0.145	0.084	0.001	0.0003
TC	rs498793	rs12939270	11:61381281	17:10012621	0.401	0.116	0.052	0.00033	0.075	0.01412	-0.105	0.084	0.001	0.00031
TC	rs3761739	rs17583177	5:74667257	11:112354976	0.148	0.07	0.076	8.08E-05	0.016	0.58467	-0.181	0.084	0.001	0.00033
TC	rs6543510	rs10962492	2:239689591	9:16561821	0.221	0.072	-0.003	0.8657	-0.007	0.81062	0.149	0.084	0.001	0.00033
TC	rs10402271	rs2075650	19:50021054	19:50087459	0.331	0.14	0.067	3.20E-05	0.211	7.03E-13	-0.094	0.088	0.001	0.00033
TC	rs2647062	rs4871598	6:32678395	8:126529172	0.167	0.28	0.000	0.99252	0.070	2.92E-05	-0.096	0.084	0.001	0.00035
TC	rs1384934	rs4806998	3:69426256	19:4747782	0.233	0.465	-0.096	0.00012	-0.044	0.00658	0.075	0.083	0.001	0.00035
TC	rs2228603	rs7252981	19:19190924	19:19553579	0.077	0.338	0.055	0.32295	0.032	0.03053	-0.144	0.085	0.001	0.00037
TC	rs9397363	rs5924611	6:151240886	23:16801918	0.054	0.422	-0.043	0.28556	0.009	0.41433	0.117	0.083	0.001	0.00037
TC	rs12811832	rs2000999	12:46662480	16:70665594	0.365	0.196	-0.069	1.76E-05	-0.011	0.6371	0.083	0.085	0.001	0.00038
TC	rs6970522	rs1800775	7:151080486	16:55552737	0.487	0.489	-0.041	0.05608	0.003	0.89167	0.064	0.085	0.001	0.00038
TC	rs11122458	rs2337901	1:228375478	2:21390756	0.218	0.371	-0.096	2.55E-05	-0.019	0.25602	0.080	0.084	0.001	0.00038
TC	rs4808199	rs7252981	19:19406099	19:19553579	0.177	0.338	0.046	0.12028	0.037	0.03218	-0.087	0.084	0.001	0.00039
TC	rs3027188	rs2715817	17:7989710	17:64994345	0.151	0.456	0.113	0.00015	0.005	0.75763	-0.090	0.081	0.001	0.0004
TC	rs2238675	rs4808199	19:19197608	19:19406099	0.132	0.177	0.023	0.41941	0.023	0.28698	-0.108	0.084	0.001	0.0004
TC	rs2001945	rs8102530	8:126547160	19:19671078	0.483	0.421	0.116	8.36E-09	0.037	0.08886	-0.064	0.085	0.001	0.0004
TC	rs2472681	rs11024739	3:121012379	11:18602419	0.302	0.263	0.050	0.00535	0.019	0.33053	-0.077	0.083	0.001	0.00041
TC	rs7306218	rs2075650	12:108532674	19:50087459	0.201	0.14	0.051	0.00498	0.186	6.27E-17	-0.113	0.087	0.001	0.00041
TC	rs215072	rs4812828	16:15993052	20:42413734	0.071	0.319	-0.080	0.02175	-0.011	0.45811	0.133	0.083	0.001	0.00041
TC	rs4144739	rs4806998	8:32089597	19:4747782	0.498	0.465	0.067	0.00145	0.054	0.0139	-0.063	0.083	0.001	0.00042
TC	rs17398765	rs4623048	2:21124256	4:41528244	0.069	0.188	0.033	0.27362	-0.008	0.63246	0.164	0.084	0.001	0.00042
TC	rs6859355	rs11551870	5:52337473	14:31695603	0.43	0.096	0.012	0.38748	-0.025	0.47505	0.108	0.084	0.001	0.00042
TC	rs1801706	rs4791489	16:55575163	17:11989001	0.178	0.383	0.142	1.16E-08	0.029	0.06307	-0.085	0.085	0.001	0.00043
TC	rs9273363	rs5110	6:32734250	11:116196844	0.267	0.082	-0.006	0.75401	0.006	0.83625	-0.128	0.084	0.001	0.00043
TC	rs2781160	rs616314	1:71868268	11:118462077	0.41	0.164	0.034	0.02818	0.079	0.00261	-0.087	0.083	0.001	0.00043
TC	rs2287622	rs657152	2:169538574	9:135129086	0.403	0.37	-0.042	0.0277	-0.011	0.57531	0.066	0.084	0.001	0.00043
TC	rs4148218	rs10246910	2:43953086	7:141660939	0.188	0.478	0.011	0.68415	0.056	0.00028	-0.081	0.085	0.001	0.00044
TC	rs6698963	rs2071437	1:228481648	12:46673186	0.462	0.216	0.014	0.39586	0.084	0.00087	-0.077	0.083	0.001	0.00046
TC	rs7761293	rs1859223	6:160890953	17:39435091	0.471	0.158	0.061	4.87E-05	0.090	0.00184	-0.086	0.084	0.001	0.00046
TC	rs10498038	rs4752805	2:215964589	11:47974931	0.058	0.247	-0.066	0.05376	-0.006	0.69451	0.158	0.083	0.001	0.00047
TC	rs1374028	rs4303611	5:58611588	17:51202987	0.104	0.061	0.081	0.00027	0.048	0.10583	-0.217	0.083	0.001	0.00047
TC	rs1531517	rs387976	19:49934013	19:50070900	0.067	0.334	0.022	0.60108	-0.036	0.01352	-0.132	0.085	0.001	0.00048
TC	rs3807370	rs3184504	7:150304247	12:110368991	0.362	0.495	-0.038	0.09263	-0.071	0.00012	0.065	0.084	0.001	0.00048
TC	rs231914	rs9900564	11:2705952	17:73889053	0.392	0.349	-0.059	0.0018	-0.075	0.00023	0.067	0.083	0.001	0.00049
TC	rs10744777	rs6602910	12:110717401	13:113564928	0.318	0.382	-0.057	0.00565	-0.028	0.1345	0.069	0.083	0.001	0.00049
TC	rs11206514	rs11751605	1:55288592	6:160883220	0.39	0.147	-0.051	0.00077	-0.038	0.15568	0.090	0.084	0.001	0.0005
TC	rs236996	rs4806998	4:88224227	19:4747782	0.395	0.465	-0.091	1.93E-05	-0.059	0.0022	0.063	0.084	0.001	0.00052
TC	rs11254269	rs6589602	10:16979846	11:116542275	0.09	0.395	-0.141	3.17E-05	0.019	0.18342	0.113	0.084	0.001	0.00053
TC	rs4299376	rs11085098	2:43926080	19:4735553	0.315	0.344	0.125	1.53E-10	0.024	0.192	-0.071	0.085	0.001	0.00053
TC	rs2149860	rs11551870	13:32526989	14:31695603	0.45	0.096	-0.024	0.08111	-0.025	0.46909	0.104	0.084	0.001	0.00053
TC	rs10246910	rs7022503	7:141660939	9:77932174	0.478	0.491	0.085	7.37E-05	0.074	0.00055	-0.062	0.083	0.001	0.00053
TC	rs4964731	rs439401	12:107581938	19:50106291	0.478	0.372	-0.066	0.00046	-0.107	1.63E-06	0.064	0.084	0.001	0.00054
TC	rs2909448	rs3171425	2:162574501	21:33590617	0.428	0.414	-0.092	3.32E-06	-0.030	0.13989	0.064	0.084	0.001	0.00054
TC	rs624612	rs629001	1:55290471	1:109640441	0.418	0.068	0.025	0.06711	0.026	0.50734	-0.124	0.084	0.001	0.00055
TC	rs17583177	rs11076174	11:112354976	16:55560647	0.07	0.082	0.001	0.9759	-0.026	0.29526	-0.221	0.084	0.001	0.00056
TC	rs4871598	rs11254269	8:126529172	10:16979846	0.28	0.09	0.016	0.29665	-0.120	4.47E-05	0.118	0.084	0.001	0.00056
TC	rs2228603	rs3794991	19:19190924	19:19471596	0.077	0.085	0.085	0.06813	-0.043	0.28361	-0.173	0.086	0.001	0.00056
TC	rs13202636	rs4812828	6:160949718	20:42413734	0.229	0.319	-0.101	1.92E-06	-0.028	0.10703	0.079	0.084	0.001	0.00056
TC	rs11752651	rs7761293	6:41668265	6:160890953	0.056	0.471	-0.073	0.1202	0.018	0.18944	0.131	0.084	0.001	0.00056
TC	rs7824367	rs12924903	8:89317031	16:30836471	0.13	0.352	0.053	0.05372	0.049	0.00138	-0.096	0.084	0.001	0.00057

S1 Table, cont'd

Lipid	V1	V2	V1|chr:bp	V2|chr:bp	V1|MAF	V2|MAF	V1|Beta	V1|pval	V2|Beta	V2|pval	Int|Beta	Full|Rsq	Diff|Rsq	lrt|pval
TC	rs180327	rs11076174	11:116128869	16:55560647	0.37	0.082	0.077	1.06E-07	0.029	0.3898	-0.116	0.085	0.001	0.00057
TC	rs10431309	rs6602910	12:7694913	13:113564928	0.236	0.382	-0.023	0.30418	-0.020	0.24073	0.076	0.084	0.001	0.00057
TC	rs2855790	rs3787268	11:128242398	20:44075138	0.185	0.209	-0.005	0.81978	0.051	0.00709	-0.098	0.084	0.001	0.00057
TC	rs2075290	rs4898398	11:116158506	23:153732140	0.072	0.092	0.075	0.00449	-0.051	0.00964	0.162	0.085	0.001	0.00058
TC	rs387976	rs405509	19:50070900	19:50100676	0.334	0.482	-0.140	8.39E-09	0.012	0.50319	0.066	0.086	0.001	0.00059
TC	rs4965843	rs1946612	15:99750749	19:46459827	0.14	0.263	0.083	0.00061	0.053	0.00146	-0.100	0.083	0.001	0.00059
TC	rs11076174	rs7220865	16:55560647	17:73889268	0.082	0.109	-0.097	0.00017	-0.049	0.03095	0.182	0.084	0.001	0.00059
TC	rs10047459	rs6003941	11:116227036	22:22568857	0.162	0.241	-0.010	0.66812	-0.029	0.09587	0.097	0.083	0.001	0.00061
TC	rs3748630	rs6708316	1:220871934	2:11854609	0.181	0.325	0.025	0.28797	0.051	0.00151	-0.084	0.083	0.001	0.00061
TC	rs978528	rs11563251	1:17222385	2:234344123	0.106	0.107	-0.039	0.09712	0.002	0.91975	0.160	0.083	0.001	0.00062
TC	rs1054564	rs7888694	19:18360815	23:20045427	0.146	0.267	-0.088	8.62E-05	-0.061	7.72E-06	0.081	0.084	0.001	0.00063
TC	rs4442034	rs2072114	7:39255911	11:61361791	0.126	0.124	-0.004	0.86759	0.019	0.39177	-0.142	0.083	0.001	0.00064
TC	rs7546522	rs11740792	1:55289301	5:142727513	0.156	0.166	0.049	0.01702	0.014	0.49373	-0.116	0.083	0.001	0.00065
TC	rs480780	rs11551870	13:32505319	14:31695603	0.387	0.096	0.019	0.19636	0.153	2.28E-06	-0.107	0.084	0.001	0.00065
TC	rs11706810	rs675	3:161642615	11:116196885	0.481	0.195	0.009	0.53844	0.068	0.01219	-0.076	0.083	0.001	0.00066
TC	rs12056414	rs7394871	8:54325439	11:61409090	0.086	0.054	0.005	0.84473	-0.086	0.00581	0.239	0.084	0.001	0.00066
TC	rs4889651	rs4791489	16:30803046	17:11989001	0.264	0.383	0.062	0.00415	0.036	0.03552	-0.072	0.083	0.001	0.00067
TC	rs2777799	rs3745551	9:106598880	19:7065288	0.116	0.375	-0.105	0.00054	-0.041	0.0059	0.099	0.083	0.001	0.00067
TC	rs3847305	rs290485	9:106697074	10:114899910	0.148	0.175	0.006	0.78574	0.030	0.11942	-0.118	0.083	0.001	0.00069
TC	rs416041	rs3171425	19:50062694	21:33590617	0.384	0.414	-0.021	0.3017	-0.024	0.21474	0.065	0.084	0.001	0.0007
TC	rs11206510	rs2738459	1:55268627	19:11099473	0.188	0.475	0.039	0.15113	0.016	0.30569	-0.078	0.083	0.001	0.00072
TC	rs630431	rs4964731	1:55299911	12:107581938	0.3	0.478	0.033	0.1594	0.021	0.22069	-0.067	0.084	0.001	0.00072
TC	rs9266831	rs416041	6:31491062	19:50062694	0.264	0.384	-0.019	0.37972	-0.005	0.78941	0.071	0.084	0.001	0.00073
TC	rs174601	rs9658605	11:61379716	12:51778766	0.361	0.071	-0.036	0.01178	0.096	0.0083	-0.122	0.084	0.001	0.00074
TC	rs2286007	rs3794241	12:841552	12:102853563	0.073	0.102	0.076	0.0049	0.004	0.85983	-0.196	0.083	0.001	0.00075
TC	rs17398765	rs4942443	2:21124256	13:31817526	0.069	0.189	0.151	7.35E-07	-0.018	0.29579	-0.152	0.084	0.001	0.00076
TC	rs12210959	rs1029621	6:6121143	7:40832234	0.26	0.203	-0.059	0.00075	-0.092	1.01E-05	0.086	0.084	0.001	0.00077
TC	rs12966382	rs5924611	18:45339438	23:16801918	0.153	0.422	0.091	0.00032	0.044	0.00041	-0.071	0.083	0.001	0.00077
TC	rs17435152	rs1801706	7:40568630	16:55575163	0.147	0.178	0.077	0.00034	0.110	1.61E-08	-0.112	0.085	0.001	0.00077
TC	rs2001945	rs1883025	8:126547160	9:106704122	0.483	0.256	0.096	6.76E-09	0.007	0.78996	-0.069	0.086	0.001	0.00079
TC	rs13004470	rs202720	2:242159756	11:49148927	0.154	0.223	0.056	0.01164	0.051	0.00398	-0.102	0.083	0.001	0.00079
TC	rs4975709	rs2385114	5:1930280	8:126516490	0.238	0.494	-0.090	0.0004	-0.003	0.84893	0.071	0.084	0.001	0.0008
TC	rs629001	rs2871865	1:109640441	15:97012419	0.068	0.113	-0.035	0.21095	0.078	0.0003	-0.189	0.084	0.001	0.00081
TC	rs2228603	rs1469713	19:19190924	19:19389806	0.077	0.354	0.049	0.38745	0.031	0.03435	-0.136	0.085	0.001	0.00081
TC	rs4299376	rs860867	2:43926080	12:42961663	0.315	0.11	0.099	1.01E-10	0.028	0.32124	-0.106	0.085	0.001	0.00082
TC	rs13219571	rs2244608	6:132320836	12:119901371	0.228	0.338	-0.063	0.0035	-0.016	0.34458	0.076	0.083	0.001	0.00083
TC	rs4938303	rs9923854	11:116090197	16:55574503	0.271	0.106	0.015	0.35276	0.000	0.98805	0.108	0.083	0.001	0.00085
TC	rs2282751	rs548638	3:50266789	11:116242303	0.131	0.223	0.008	0.73338	0.006	0.71465	-0.106	0.083	0.001	0.00086
TC	rs6657811	rs6073418	1:109608806	20:42434004	0.13	0.349	-0.195	2.53E-12	-0.040	0.00878	0.094	0.086	0.001	0.00087
TC	rs754523	rs387976	2:21165196	19:50070900	0.317	0.334	0.054	0.00519	-0.105	2.56E-08	0.068	0.088	0.001	0.00087
TC	rs13182800	rs616314	5:142781673	11:118462077	0.204	0.164	-0.068	0.00032	-0.032	0.1352	0.101	0.083	0.001	0.00088
TC	rs6433688	rs11254269	2:178245922	10:16979846	0.141	0.09	0.053	0.00739	-0.011	0.67598	-0.154	0.084	0.001	0.00088
TC	rs17730649	rs11254269	8:126534487	10:16979846	0.413	0.09	0.011	0.42698	-0.138	4.87E-05	0.104	0.084	0.001	0.00089
TC	rs868690	rs2241201	12:53327542	12:108476515	0.243	0.274	0.090	5.92E-06	0.079	1.67E-05	-0.078	0.084	0.001	0.0009
TC	rs845555	rs797205	7:55214828	13:32659410	0.447	0.194	-0.001	0.93792	-0.086	0.00093	0.076	0.083	0.001	0.00091
TC	rs2287622	rs16924332	2:169538574	12:21865495	0.403	0.347	0.051	0.0056	0.055	0.00736	-0.064	0.083	0.001	0.00092
TC	rs10498038	rs2072583	2:215964589	3:8750457	0.058	0.255	0.078	0.02531	0.004	0.80208	-0.149	0.083	0.001	0.00092
TC	rs8102912	rs11085258	19:11066975	19:19150830	0.223	0.382	-0.134	5.68E-09	-0.040	0.01386	0.073	0.085	0.001	0.00093
TC	rs2254884	rs2244608	9:106621570	12:119901371	0.293	0.338	-0.015	0.44562	-0.022	0.21773	0.069	0.083	0.001	0.00093
TC	rs693	rs289716	2:21085700	16:55566877	0.496	0.316	0.064	0.00025	0.004	0.86474	0.063	0.090	0.001	0.00093
TC	rs1578275	rs7260463	1:241747065	19:50506700	0.173	0.328	0.069	0.00492	0.056	0.00044	-0.083	0.082	0.001	0.00094
TC	rs2228671	rs11085258	19:11071912	19:19150830	0.12	0.382	-0.189	2.22E-10	-0.030	0.041	0.094	0.085	0.001	0.00094
TC	rs7816340	rs11024739	8:26721504	11:18602419	0.116	0.263	-0.038	0.15203	-0.052	0.00115	0.103	0.083	0.001	0.00094
TC	rs6761276	rs3793891	2:113548783	10:112254954	0.42	0.082	0.023	0.09553	0.108	0.00205	-0.107	0.083	0.001	0.00094

S1 Table, cont'd

Lipid	V1	V2	V1|chr:bp	V2|chr:bp	V1|MAF	V2|MAF	V1|Beta	V1|pval	V2|Beta	V2|pval	Int|Beta	Full|Rsq	Diff|Rsq	lrt|pval
TC	rs2301128	rs5110	11:113673209	11:116196844	0.195	0.082	-0.043	0.01472	-0.118	3.08E-05	0.133	0.084	0.001	0.00094
TC	rs4144739	rs2066716	8:32089597	9:106608526	0.498	0.09	0.028	0.05426	0.161	3.26E-05	-0.105	0.083	0.001	0.00094
TC	rs4148809	rs6003941	7:86941199	22:22568857	0.459	0.241	-0.052	0.00173	-0.061	0.01277	0.070	0.082	0.001	0.00096
TC	rs17655652	rs2965101	7:44547516	19:49929652	0.316	0.327	0.029	0.12675	0.033	0.07953	-0.068	0.083	0.001	0.00096
TC	rs585362	rs1029621	1:109591318	7:40832234	0.154	0.203	-0.138	3.33E-10	-0.078	2.52E-05	0.103	0.086	0.001	0.00097
TC	rs2777784	rs2254819	9:106700880	9:106706235	0.382	0.466	0.046	0.0581	0.020	0.32393	-0.061	0.084	0.001	0.00098
TC	rs12203834	rs6663	6:109082255	12:108370986	0.114	0.252	0.043	0.09347	0.063	0.00012	-0.106	0.083	0.001	0.00098
TC	rs180327	rs572410	11:116128869	15:56528676	0.37	0.238	0.024	0.15976	-0.024	0.2747	0.072	0.085	0.001	0.00098
TC	rs2280801	rs387976	6:31700043	19:50070900	0.056	0.334	0.146	0.00023	-0.045	0.00181	-0.138	0.085	0.001	0.00099
TC	rs13004470	rs657152	2:242159756	9:135129086	0.154	0.37	-0.052	0.04693	0.016	0.29146	0.085	0.084	0.001	0.001
TG	rs4938303	rs180327	11:116090197	11:116128869	0.271	0.371	-0.009	0.40801	-0.009	0.28271	0.093	0.227	0.006	1.25E-21
TG	rs2075295	rs6589568	11:116133611	11:116175948	0.264	0.21	0.056	3.86E-09	0.119	1.28E-24	-0.100	0.220	0.005	4.45E-19
TG	rs180327	rs10750097	11:116128869	11:116169250	0.371	0.209	0.002	0.78352	0.013	0.30488	0.082	0.229	0.003	3.09E-14
TG	rs180327	rs2075295	11:116128869	11:116133611	0.371	0.264	0.022	0.00688	-0.025	0.0108	0.073	0.222	0.003	8.87E-13
TG	rs180327	rs6589568	11:116128869	11:116175948	0.371	0.21	0.028	0.00037	-0.004	0.73693	0.068	0.223	0.002	2.69E-10
TG	rs11216129	rs10750097	11:116125466	11:116169250	0.125	0.209	-0.013	0.34377	0.151	2.67E-58	-0.086	0.230	0.002	2.14E-09
TG	rs180327	rs689243	11:116128869	11:116227903	0.371	0.368	0.008	0.38632	-0.010	0.27804	0.054	0.221	0.002	3.10E-09
TG	rs481843	rs689243	11:116031077	11:116227903	0.082	0.368	0.007	0.71421	0.015	0.03161	0.095	0.223	0.002	4.71E-09
TG	rs486394	rs689243	11:116031532	11:116227903	0.289	0.368	-0.009	0.3893	0.006	0.47993	0.054	0.219	0.002	2.87E-08
TG	rs11216129	rs5142	11:116125466	11:116207060	0.125	0.09	-0.001	0.9549	0.126	4.17E-24	-0.116	0.220	0.002	3.10E-08
TG	rs2075295	rs5142	11:116133611	11:116207060	0.264	0.09	0.037	1.01E-06	0.138	1.01E-22	-0.090	0.220	0.002	6.61E-08
TG	rs180327	rs5142	11:116128869	11:116207060	0.371	0.09	0.034	1.49E-06	-0.003	0.85395	0.079	0.223	0.002	2.87E-07
TG	rs180327	rs618923	11:116128869	11:116159369	0.371	0.251	0.144	2.56E-56	-0.060	3.78E-05	-0.053	0.231	0.001	3.74E-07
TG	rs180327	rs4938315	11:116128869	11:116236415	0.371	0.116	0.037	3.08E-07	-0.006	0.70149	0.068	0.221	0.001	4.48E-07
TG	rs2075295	rs1263173	11:116133611	11:116186218	0.264	0.278	0.050	4.17E-07	0.050	1.14E-07	-0.051	0.215	0.001	9.28E-07
TG	rs180327	rs675	11:116128869	11:116196885	0.371	0.194	0.097	3.73E-33	-0.004	0.791	-0.054	0.222	0.001	1.39E-06
TG	rs481843	rs4938303	11:116031077	11:116090197	0.082	0.271	0.011	0.5963	0.049	3.27E-10	0.078	0.223	0.001	5.02E-06
TG	rs618923	rs7396851	11:116159369	11:116189374	0.251	0.136	-0.025	0.00227	0.092	3.84E-14	-0.063	0.218	0.001	5.44E-06
TG	rs486394	rs4938303	11:116031532	11:116090197	0.289	0.271	-0.006	0.4946	0.042	2.03E-05	0.047	0.220	0.001	5.75E-06
TG	rs180327	rs10047459	11:116128869	11:116227036	0.371	0.163	0.037	7.03E-07	-0.008	0.52655	0.053	0.220	0.001	5.79E-06
TG	rs405509	rs12721046	19:50100676	19:50113094	0.486	0.144	-0.033	3.56E-06	-0.009	0.6038	0.058	0.216	0.001	6.99E-06
TG	rs213950	rs8042050	7:116986769	15:61142924	0.409	0.129	0.024	0.00052	0.069	6.64E-07	-0.058	0.215	0.001	7.76E-06
TG	rs180327	rs548638	11:116128869	11:116242303	0.371	0.222	0.092	1.76E-29	-0.011	0.37047	-0.046	0.222	0.001	9.61E-06
TG	rs180327	rs6589602	11:116128869	11:116542275	0.371	0.396	0.021	0.03403	-0.001	0.92137	0.040	0.220	0.001	1.10E-05
TG	rs481843	rs6589602	11:116031077	11:116542275	0.082	0.396	0.034	0.09391	0.014	0.04301	0.070	0.222	0.001	1.13E-05
TG	rs6589568	rs7396851	11:116175948	11:116189374	0.21	0.136	0.015	0.11474	-0.010	0.47636	0.059	0.217	0.001	1.38E-05
TG	rs180327	rs2306473	11:116128869	11:116603162	0.371	0.164	0.038	2.99E-07	-0.008	0.5308	0.052	0.220	0.001	1.50E-05
TG	rs486394	rs625145	11:116031532	11:116233146	0.289	0.199	0.016	0.05199	-0.008	0.45671	0.051	0.217	0.001	1.61E-05
TG	rs285	rs7013777	8:19859469	8:19922636	0.476	0.46	0.002	0.85424	-0.003	0.78346	-0.037	0.218	0.001	1.68E-05
TG	rs11711437	rs2980883	3:172209057	8:126543587	0.137	0.153	-0.012	0.25584	0.008	0.42253	0.075	0.215	0.001	1.73E-05
TG	rs1538843	rs11571340	9:77791088	17:6848943	0.084	0.067	-0.039	0.00083	-0.036	0.00625	0.136	0.215	0.001	1.84E-05
TG	rs17151244	rs4521758	8:10066862	8:48904123	0.059	0.114	0.044	0.0031	0.031	0.00259	-0.121	0.214	0.001	1.87E-05
TG	rs1996025	rs1211375	4:149525557	16:180281	0.097	0.363	-0.047	0.00172	0.007	0.29932	0.063	0.215	0.001	2.22E-05
TG	rs7859486	rs1864163	9:77893732	16:55554734	0.181	0.253	0.052	3.60E-07	0.028	0.00078	-0.054	0.215	0.001	2.54E-05
TG	rs1211375	rs2238675	16:180281	19:19197608	0.363	0.133	0.034	2.02E-06	-0.004	0.76715	-0.055	0.216	0.001	2.75E-05
TG	rs4846908	rs609526	1:228351679	1:228375529	0.465	0.412	0.027	0.00491	0.042	2.92E-05	-0.037	0.214	0.001	2.79E-05
TG	rs898034	rs4652	2:27944324	14:54674789	0.209	0.433	-0.067	1.47E-08	0.005	0.48446	0.044	0.216	0.001	3.14E-05
TG	rs4147542	rs2652324	4:100487576	5:156086091	0.263	0.181	0.030	0.00031	0.006	0.53813	-0.052	0.215	0.001	3.28E-05
TG	rs157580	rs405509	19:50087106	19:50100676	0.389	0.486	0.010	0.45708	0.037	0.00012	-0.037	0.215	0.001	4.21E-05
TG	rs6589568	rs1263173	11:116175948	11:116186218	0.21	0.278	0.007	0.5841	-0.023	0.01897	0.046	0.216	0.001	4.50E-05
TG	rs228697	rs4766602	1:7810166	12:108374893	0.107	0.432	0.053	0.00066	0.016	0.01938	-0.058	0.214	0.001	4.92E-05
TG	rs1167998	rs1211375	1:62704220	16:180281	0.335	0.363	-0.003	0.72594	0.045	4.10E-07	-0.038	0.216	0.001	5.04E-05
TG	rs698	rs2305654	4:100479812	15:39924269	0.402	0.336	-0.041	5.34E-06	-0.024	0.01504	0.038	0.212	0.001	5.04E-05
TG	rs4784339	rs730079	16:52597179	19:18764495	0.39	0.381	0.033	0.00031	0.030	0.00163	-0.036	0.214	0.001	5.98E-05

S1 Table, cont'd

Lipid	V1	V2	V1|chr:bp	V2|chr:bp	V1|MAF	V2|MAF	V1|Beta	V1|pval	V2|Beta	V2|pval	Int|Beta	Full|Rsq	Diff|Rsq	lrt|pval
TG	rs486394	rs6589602	11:116031532	11:116542275	0.289	0.396	0.004	0.68693	0.010	0.23498	0.039	0.218	0.001	6.77E-05
TG	rs486394	rs2075295	11:116031532	11:116133611	0.289	0.264	0.015	0.0845	-0.006	0.55481	0.042	0.216	0.001	7.32E-05
TG	rs2300586	rs140700	7:44185381	17:25567515	0.151	0.088	-0.004	0.70401	-0.033	0.0073	0.083	0.214	0.001	9.13E-05
TG	rs618923	rs5142	11:116159369	11:116207060	0.251	0.09	-0.024	0.00142	0.116	1.82E-18	-0.073	0.220	0.001	9.37E-05
TG	rs2075295	rs10047459	11:116133611	11:116227036	0.264	0.163	0.035	2.97E-05	0.066	6.51E-09	-0.050	0.216	0.001	9.62E-05
TG	rs486394	rs618923	11:116031532	11:116159369	0.289	0.251	0.064	4.17E-13	-0.017	0.07095	-0.042	0.218	0.001	0.0001
TG	rs2227667	rs213950	7:100561469	7:116986769	0.21	0.409	0.041	0.00029	0.027	0.00039	-0.041	0.214	0.001	0.00012
TG	rs4652	rs10483801	14:54674789	14:67186759	0.433	0.183	0.008	0.29179	-0.037	0.00297	0.043	0.215	0.001	0.00012
TG	rs1801700	rs6589602	2:21099318	11:116542275	0.051	0.396	0.085	6.09E-05	0.043	7.42E-11	-0.078	0.216	0.001	0.00013
TG	rs4938303	rs689243	11:116090197	11:116227903	0.271	0.368	0.037	0.00195	-0.010	0.28716	0.038	0.219	0.001	0.00013
TG	rs2238675	rs7252981	19:19197608	19:19553579	0.133	0.337	-0.002	0.87118	0.019	0.0113	-0.049	0.215	0.001	0.00014
TG	rs4871594	rs2980883	8:126511247	8:126543587	0.477	0.153	0.016	0.02214	0.075	6.57E-07	-0.045	0.214	0.001	0.00014
TG	rs6589568	rs2306473	11:116175948	11:116603162	0.21	0.164	0.066	5.25E-14	0.060	1.45E-09	-0.056	0.218	0.001	0.00015
TG	rs12048208	rs1211375	1:62827868	16:180281	0.125	0.363	0.010	0.47464	0.032	5.94E-06	-0.050	0.215	0.001	0.00017
TG	rs4849148	rs12501467	2:113545993	4:5794880	0.064	0.135	0.041	0.004	0.006	0.55624	-0.096	0.214	0.001	0.00017
TG	rs180327	rs1263173	11:116128869	11:116186218	0.371	0.278	0.034	5.17E-05	-0.010	0.31397	0.037	0.219	0.001	0.00017
TG	rs12686004	rs7359336	9:106693247	16:68290961	0.116	0.425	0.030	0.04523	0.002	0.73635	-0.051	0.214	0.001	0.00018
TG	rs1889548	rs2980884	1:94275785	8:126543538	0.312	0.37	0.031	0.00124	0.049	2.84E-08	-0.036	0.214	0.001	0.00018
TG	rs4147542	rs4081918	4:100487576	19:50065579	0.263	0.096	0.022	0.00306	0.001	0.91153	-0.060	0.214	0.001	0.00018
TG	rs7837316	rs2995991	8:10225402	13:40049134	0.33	0.4	0.023	0.04858	0.008	0.33172	-0.035	0.214	0.001	0.00019
TG	rs12686004	rs12827659	9:106693247	12:26236473	0.116	0.056	-0.002	0.87265	0.000	0.99432	-0.110	0.214	0.001	0.0002
TG	rs6589568	rs4938315	11:116175948	11:116236415	0.21	0.116	0.018	0.05702	0.006	0.68406	0.055	0.217	0.001	0.00021
TG	rs10750097	rs7396851	11:116169250	11:116189374	0.209	0.136	0.077	4.26E-17	-0.014	0.27518	0.051	0.225	0.001	0.00021
TG	rs4394275	rs1966435	6:31426156	16:52588027	0.247	0.439	-0.029	0.01394	-0.027	0.00045	0.037	0.214	0.001	0.00021
TG	rs13277738	rs6142777	8:18285787	20:60492195	0.152	0.193	-0.006	0.59066	-0.005	0.60369	0.057	0.214	0.001	0.00022
TG	rs4663965	rs2107131	2:234315343	17:33160802	0.448	0.329	0.022	0.011	0.021	0.03968	-0.034	0.214	0.001	0.00022
TG	rs2127565	rs2489040	8:16030930	10:102321288	0.131	0.219	0.011	0.30809	0.030	0.00039	-0.056	0.214	0.001	0.00024
TG	rs6984111	rs3729989	8:10249198	11:47326617	0.155	0.128	0.012	0.21118	0.023	0.03081	-0.064	0.214	0.001	0.00028
TG	rs17269264	rs1800775	15:56526052	16:55552737	0.482	0.485	-0.021	0.04192	-0.040	0.00011	0.031	0.214	0.001	0.00029
TG	rs2673148	rs236996	2:226883227	4:88224227	0.484	0.399	-0.005	0.56338	-0.052	6.41E-07	0.031	0.215	0.001	0.00031
TG	rs2228603	rs7252981	19:19190924	19:19553579	0.077	0.337	0.019	0.46659	0.019	0.00722	-0.070	0.216	0.001	0.00032
TG	rs7586601	rs4361084	2:27438170	2:27915900	0.434	0.263	-0.027	0.00084	0.053	1.34E-06	-0.036	0.217	0.001	0.00033
TG	rs6984111	rs12575157	8:10249198	11:47229322	0.155	0.14	0.012	0.22079	0.013	0.19766	-0.061	0.214	0.001	0.00034
TG	rs7586601	rs17410962	2:27438170	8:19892360	0.434	0.125	-0.059	5.37E-17	-0.117	1.27E-15	0.047	0.220	0.001	0.00036
TG	rs2227667	rs2128806	7:100561469	11:103531675	0.21	0.482	-0.029	0.02168	-0.017	0.02841	0.038	0.214	0.001	0.00036
TG	rs3729989	rs5747997	11:47326617	22:17504375	0.128	0.487	-0.043	0.0061	-0.021	0.00214	0.046	0.214	0.001	0.00036
TG	rs4519913	rs7942717	5:147452004	11:61403864	0.487	0.085	0.019	0.00335	0.097	3.64E-07	-0.056	0.215	0.001	0.00036
TG	rs638820	rs4251520	1:110011429	12:42461605	0.498	0.112	0.005	0.4241	0.034	0.0394	-0.049	0.214	0.001	0.00037
TG	rs6601548	rs12686004	8:10925506	9:106693247	0.088	0.116	0.028	0.02424	0.002	0.85494	-0.086	0.214	0.001	0.00037
TG	rs4148324	rs2107131	2:234337461	17:33160802	0.33	0.329	0.025	0.00591	0.014	0.12012	-0.035	0.214	0.001	0.00037
TG	rs17151244	rs11594681	8:10066862	10:114873312	0.059	0.366	-0.034	0.07249	-0.017	0.01119	0.066	0.214	0.001	0.00039
TG	rs2268569	rs6601548	7:44193545	8:10925506	0.08	0.088	0.032	0.00969	0.025	0.03904	-0.100	0.214	0.001	0.00039
TG	rs1801700	rs1263173	2:21099318	11:116186218	0.051	0.278	0.067	0.00029	0.031	2.26E-05	-0.079	0.215	0.001	0.00039
TG	rs481843	rs2075295	11:116031077	11:116133611	0.082	0.264	0.086	8.66E-10	0.012	0.09347	0.063	0.221	0.001	0.00041
TG	rs270	rs6586891	8:19857956	8:19958878	0.159	0.347	0.053	8.27E-06	0.056	7.81E-14	-0.043	0.217	0.001	0.00042
TG	rs12257915	rs2489040	10:90982709	10:102321288	0.421	0.219	0.006	0.44172	0.046	6.35E-05	-0.037	0.214	0.001	0.00042
TG	rs6547735	rs4652	2:27685111	14:54674789	0.205	0.433	-0.067	1.85E-08	0.008	0.2978	0.037	0.216	0.001	0.00043
TG	rs162029	rs17704764	5:7918527	5:147468847	0.173	0.221	-0.049	1.40E-06	-0.025	0.00363	0.049	0.215	0.001	0.00043
TG	rs13250578	rs838896	8:11651714	12:123835752	0.142	0.333	-0.026	0.0444	-0.014	0.0694	0.045	0.214	0.001	0.00044
TG	rs2074754	rs2286276	7:72529690	7:72625290	0.481	0.283	-0.004	0.61307	-0.075	8.83E-12	0.034	0.217	0.001	0.00045
TG	rs7816713	rs2072114	8:10224199	11:61361791	0.477	0.125	0.003	0.68996	0.080	2.16E-07	-0.045	0.215	0.001	0.00045
TG	rs12358632	rs133029	10:84101439	22:36906261	0.077	0.102	0.041	0.0011	-0.007	0.50661	-0.092	0.214	0.001	0.00046
TG	rs1736093	rs6987702	8:11704905	8:126573908	0.208	0.27	-0.029	0.00691	0.002	0.85108	0.041	0.214	0.001	0.00046
TG	rs2409814	rs11071428	8:11643561	15:57402133	0.443	0.146	0.021	0.03294	0.050	0.00028	-0.043	0.214	0.001	0.00047

S1 Table, cont'd

Lipid	V1	V2	V1|chr:bp	V2|chr:bp	V1|MAF	V2|MAF	V1|Beta	V1|pval	V2|Beta	V2|pval	Int|Beta	Full|Rsq	Diff|Rsq	lrt|pval
TG	rs9320208	rs799168	6:107659173	7:72688800	0.227	0.221	0.032	0.00034	-0.018	0.05577	-0.044	0.216	0.001	0.00047
TG	rs17138476	rs2235133	17:33149718	21:45145600	0.164	0.459	-0.047	0.00052	-0.005	0.50253	0.041	0.214	0.001	0.00047
TG	rs7522692	rs4846904	1:170703458	1:228346033	0.21	0.193	-0.005	0.55357	-0.005	0.58087	0.047	0.214	0.001	0.00047
TG	rs7396851	rs625145	11:116189374	11:116233146	0.136	0.199	0.070	2.40E-10	0.039	9.44E-06	-0.053	0.216	0.001	0.00048
TG	rs1808602	rs2129732	3:30665428	17:40553119	0.46	0.227	-0.008	0.3298	-0.039	0.00116	0.036	0.214	0.001	0.00048
TG	rs10778520	rs140700	12:105889003	17:25567515	0.429	0.088	0.014	0.04415	0.035	0.03522	-0.053	0.213	0.001	0.00048
TG	rs9431818	rs6444435	1:228484622	3:191786499	0.492	0.338	-0.031	0.00037	-0.019	0.07692	0.031	0.214	0.001	0.00048
TG	rs4135247	rs6560490	3:12371588	9:77846158	0.416	0.33	-0.010	0.23679	-0.024	0.01917	0.032	0.214	0.001	0.00049
TG	rs486394	rs17110747	11:116031532	12:70712221	0.289	0.138	0.051	2.82E-11	0.021	0.07739	-0.048	0.216	0.001	0.0005
TG	rs804282	rs1263173	8:11649152	11:116186218	0.461	0.278	-0.017	0.05208	-0.008	0.4637	0.034	0.214	0.001	0.00051
TG	rs2227667	rs253	7:100561469	8:19855697	0.21	0.451	0.041	0.00066	0.000	0.96924	-0.037	0.214	0.001	0.00051
TG	rs1358753	rs1576817	6:161010560	10:90987444	0.137	0.443	-0.048	0.00079	-0.002	0.77691	0.043	0.214	0.001	0.00051
TG	rs2925949	rs4519913	4:37586818	5:147452004	0.402	0.487	-0.019	0.06641	-0.013	0.142	0.030	0.214	0.001	0.00051
TG	rs10892306	rs11673139	11:118256536	19:50074877	0.063	0.093	0.009	0.52187	-0.020	0.06836	-0.103	0.214	0.001	0.00051
TG	rs923046	rs4521758	8:10010205	8:48904123	0.072	0.114	0.032	0.01693	0.029	0.00468	-0.093	0.214	0.001	0.00052
TG	rs10489626	rs2777802	1:67565759	9:106609158	0.175	0.235	0.027	0.00776	0.005	0.58774	-0.046	0.214	0.001	0.00052
TG	rs274558	rs2268569	5:131749073	7:44193545	0.396	0.08	0.004	0.58034	-0.031	0.06956	0.057	0.214	0.001	0.00053
TG	rs11216129	rs4938315	11:116125466	11:116236415	0.125	0.116	-0.006	0.5988	0.081	1.26E-13	-0.066	0.216	0.001	0.00054
TG	rs2230593	rs663214	3:49915082	15:41901074	0.068	0.282	-0.045	0.00469	0.009	0.21126	0.065	0.214	0.001	0.00054
TG	rs4251520	rs16960344	12:42461605	17:62319516	0.112	0.104	-0.030	0.0049	0.008	0.46182	0.078	0.214	0.001	0.00055
TG	rs4150018	rs13392177	1:240119244	2:218821201	0.428	0.495	-0.033	0.00146	-0.026	0.00718	0.030	0.214	0.001	0.00056
TG	rs1889548	rs2235108	1:94275785	8:126517971	0.312	0.301	0.024	0.00623	0.037	5.12E-05	-0.035	0.214	0.001	0.00057
TG	rs10489626	rs2030910	1:67565759	7:113764950	0.175	0.395	-0.026	0.03413	-0.003	0.72924	0.039	0.214	0.001	0.00058
TG	rs4655537	rs482548	1:65831389	11:61389758	0.354	0.094	-0.010	0.14678	-0.057	0.00017	0.053	0.214	0.001	0.00059
TG	rs11986942	rs9912576	8:19911725	17:37572260	0.309	0.129	-0.051	8.93E-12	0.040	0.00129	-0.047	0.219	0.001	0.00059
TG	rs486394	rs3751667	11:116031532	16:944555	0.289	0.227	0.055	4.27E-11	0.015	0.11201	-0.038	0.216	0.001	0.00061
TG	rs3860064	rs3729989	4:37577548	11:47326617	0.223	0.128	0.004	0.62701	0.027	0.02019	-0.052	0.214	0.001	0.00062
TG	rs2734335	rs7942717	6:32001923	11:61403864	0.488	0.085	-0.017	0.04006	-0.007	0.69129	0.052	0.214	0.001	0.00062
TG	rs10483801	rs12708967	14:67186759	16:55550712	0.183	0.182	-0.018	0.06038	0.000	0.99752	0.047	0.214	0.001	0.00063
TG	rs261341	rs2075650	15:56518859	19:50087459	0.295	0.145	0.026	0.0005	0.092	7.27E-15	-0.045	0.217	0.001	0.00064
TG	rs6535580	rs4632263	4:149241660	19:19073298	0.216	0.281	-0.016	0.09709	-0.040	3.14E-06	0.039	0.214	0.001	0.00065
TG	rs9853387	rs157580	3:137521678	19:50087106	0.337	0.389	-0.010	0.28233	-0.047	1.06E-07	0.031	0.215	0.001	0.00065
TG	rs9320208	rs164632	6:107659173	19:4090849	0.227	0.266	0.035	0.00019	0.003	0.74912	-0.039	0.214	0.001	0.00067
TG	rs2644627	rs7942717	8:26782558	11:61403864	0.445	0.085	0.006	0.39958	0.092	2.92E-07	-0.054	0.213	0.001	0.00067
TG	rs16849671	rs4871598	3:167010073	8:126529172	0.082	0.279	-0.006	0.68365	0.015	0.03523	0.059	0.215	0.001	0.00068
TG	rs12048208	rs4782812	1:62827868	16:82121112	0.125	0.055	-0.037	0.00014	-0.060	8.65E-05	0.093	0.215	0.001	0.00069
TG	rs611841	rs6034984	1:228376104	20:1813507	0.157	0.092	0.008	0.37083	-0.036	0.00395	0.068	0.214	0.001	0.0007
TG	rs693	rs2306473	2:21085700	11:116603162	0.5	0.164	-0.006	0.42969	0.075	9.34E-08	-0.039	0.216	0.001	0.0007
TG	rs7933164	rs9897362	11:4050526	17:17397471	0.128	0.057	-0.024	0.01296	0.005	0.72874	0.093	0.214	0.001	0.00071
TG	rs4619879	rs12521436	4:23443974	5:142797800	0.364	0.172	-0.009	0.22478	0.002	0.88447	0.040	0.214	0.001	0.00071
TG	rs2227672	rs213950	7:100562406	7:116986769	0.13	0.409	0.048	0.00049	0.021	0.00268	-0.044	0.214	0.001	0.00071
TG	rs13501	rs1864163	6:32901501	16:55554734	0.32	0.253	0.030	0.00063	0.031	0.00129	-0.036	0.214	0.001	0.00071
TG	rs709822	rs838896	8:11739722	12:123835752	0.289	0.333	-0.017	0.11509	-0.020	0.02133	0.034	0.214	0.001	0.00072
TG	rs3024346	rs9273363	6:6248245	6:32734250	0.068	0.269	-0.017	0.27214	-0.030	0.00039	0.062	0.214	0.001	0.00072
TG	rs17199228	rs8033972	2:203126028	15:62312033	0.105	0.274	-0.011	0.41848	-0.034	6.71E-06	0.052	0.215	0.001	0.00073
TG	rs4251520	rs2301157	12:42461605	13:102496364	0.112	0.444	0.026	0.0922	-0.004	0.61175	-0.045	0.214	0.001	0.00073
TG	rs1794066	rs5167	2:113602821	19:50140305	0.399	0.353	0.023	0.01	0.037	0.00017	-0.031	0.214	0.001	0.00073
TG	rs4835490	rs4081918	4:149309776	19:50065579	0.191	0.096	-0.027	0.00142	-0.054	1.57E-05	0.063	0.214	0.001	0.00073
TG	rs263	rs164632	8:19857092	19:4090849	0.179	0.266	-0.076	1.61E-13	-0.030	0.00022	0.043	0.217	0.001	0.00075
TG	rs1481031	rs6034984	18:59003065	20:1813507	0.328	0.092	-0.001	0.85939	0.021	0.15658	-0.053	0.214	0.001	0.00076
TG	rs4674309	rs2720305	2:219022817	12:48438244	0.181	0.307	-0.029	0.00805	0.005	0.54146	0.041	0.214	0.001	0.00076
TG	rs2971672	rs140700	7:44172431	17:25567515	0.374	0.088	0.006	0.4006	-0.048	0.00234	0.054	0.214	0.001	0.00076
TG	rs675	rs12708967	11:116196885	16:55550712	0.194	0.182	-0.042	5.35E-06	0.000	0.96945	0.047	0.214	0.001	0.00076
TG	rs3773967	rs1883025	3:191787712	9:106704122	0.195	0.254	-0.011	0.2856	-0.025	0.00399	0.042	0.213	0.001	0.00078

S1 Table, cont'd

Lipid	V1	V2	V1|chr:bp	V2|chr:bp	V1|MAF	V2|MAF	V1|Beta	V1|pval	V2|Beta	V2|pval	Int|Beta	Full|Rsq	Diff|Rsq	lrt|pval
TG	rs2075295	rs10750097	11:116133611	11:116169250	0.264	0.209	-0.012	0.22809	0.146	1.90E-32	-0.038	0.226	0.001	0.00078
TG	rs1889548	rs2980885	1:94275785	8:126543488	0.312	0.231	0.020	0.01368	0.054	7.02E-08	-0.037	0.215	0.001	0.00079
TG	rs12521436	rs6511040	5:142797800	19:19532121	0.172	0.402	0.063	3.11E-07	0.015	0.0412	-0.038	0.215	0.001	0.0008
TG	rs2235108	rs17730649	8:126517971	8:126534487	0.301	0.41	0.041	0.00011	0.039	4.54E-06	-0.032	0.215	0.001	0.0008
TG	rs4835490	rs2834190	4:149309776	21:33614881	0.191	0.283	-0.038	0.00023	-0.033	5.19E-05	0.041	0.214	0.001	0.00081
TG	rs610293	rs9912576	11:66066956	17:37572260	0.316	0.129	0.024	0.00129	0.040	0.00127	-0.046	0.214	0.001	0.00082
TG	rs10750097	rs11045241	11:116169250	12:20474288	0.209	0.328	0.075	1.34E-12	-0.037	4.92E-06	0.038	0.225	0.001	0.00082
TG	rs17704764	rs2256965	5:147468847	6:31663109	0.221	0.422	0.021	0.06651	0.021	0.01818	-0.035	0.214	0.001	0.00083
TG	rs17704764	rs7942717	5:147468847	11:61403864	0.221	0.085	-0.020	0.01295	0.014	0.31549	0.062	0.215	0.001	0.00083
TG	rs4632602	rs9886813	4:164486579	9:77854225	0.111	0.239	-0.012	0.33781	-0.007	0.39851	0.053	0.214	0.001	0.00084
TG	rs4148324	rs11071428	2:234337461	15:57402133	0.33	0.146	-0.010	0.16541	-0.017	0.16314	0.043	0.214	0.001	0.00085
TG	rs763317	rs4652	7:55062691	14:54674789	0.479	0.433	0.017	0.08501	0.051	7.20E-07	-0.029	0.215	0.001	0.00085
TG	rs4938303	rs7396851	11:116090197	11:116189374	0.271	0.136	0.060	1.51E-13	-0.008	0.5697	0.044	0.219	0.001	0.00085
TG	rs1263173	rs625145	11:116186218	11:116233146	0.278	0.199	0.037	1.94E-05	0.047	1.35E-05	-0.039	0.215	0.001	0.00085
TG	rs481843	rs2075650	11:116031077	19:50087459	0.082	0.145	0.095	1.46E-13	0.052	3.70E-08	0.073	0.224	0.001	0.00087
TG	rs1921752	rs231348	7:40763143	11:2630257	0.371	0.116	-0.019	0.0071	-0.023	0.10086	0.046	0.214	0.001	0.00088
TG	rs9886813	rs976165	9:77854225	20:834856	0.239	0.263	0.025	0.00687	0.002	0.79594	-0.038	0.214	0.001	0.0009
TG	rs4303700	rs17211510	2:202954656	6:32710408	0.234	0.264	-0.024	0.00917	0.004	0.64775	0.039	0.214	0.001	0.0009
TG	rs3819197	rs2409814	4:100419532	8:11643561	0.244	0.443	-0.009	0.41818	-0.008	0.46349	0.033	0.214	0.001	0.0009
TG	rs486394	rs675	11:116031532	11:116196885	0.289	0.194	0.058	6.60E-12	-0.005	0.64186	-0.038	0.217	0.001	0.00091
TG	rs4271002	rs6490700	8:18292548	13:21629267	0.119	0.441	0.054	0.00033	0.019	0.00666	-0.044	0.214	0.001	0.00092
TG	rs13277738	rs2777799	8:18285787	9:106598880	0.152	0.115	0.030	0.00172	0.001	0.93382	-0.063	0.214	0.001	0.00092
TG	rs9320208	rs16967611	6:107659173	17:37655093	0.227	0.295	0.036	0.00023	0.021	0.01161	-0.037	0.214	0.001	0.00094
TG	rs3821445	rs838896	3:137485499	12:123835752	0.201	0.333	-0.048	8.40E-06	-0.016	0.05114	0.037	0.214	0.001	0.00094
TG	rs4810479	rs915943	20:43978455	23:153280690	0.251	0.198	0.026	0.00114	0.003	0.67197	-0.034	0.212	0.001	0.00094
TG	rs2980883	rs890844	8:126543587	16:24414624	0.153	0.352	0.055	6.53E-06	0.019	0.00912	-0.041	0.213	0.001	0.00095
TG	rs28927680	rs12610605	11:116124283	19:50062678	0.07	0.17	0.103	3.23E-13	-0.024	0.00567	0.072	0.220	0.001	0.00095
TG	rs537160	rs17151244	6:32024379	8:10066862	0.322	0.059	-0.008	0.47006	-0.027	0.13915	0.063	0.214	0.001	0.00096
TG	rs2353082	rs10426094	7:72551104	19:7156240	0.269	0.23	0.035	5.68E-05	-0.003	0.76091	-0.038	0.214	0.001	0.00096
TG	rs9804646	rs5142	11:116170289	11:116207060	0.08	0.09	-0.012	0.31977	0.105	2.22E-19	-0.088	0.219	0.001	0.00097
TG	rs10750097	rs5142	11:116169250	11:116207060	0.209	0.09	0.075	1.86E-17	0.005	0.78418	0.055	0.226	0.001	0.00097
TG	rs9369425	rs12772424	6:43918952	10:114870541	0.29	0.406	0.019	0.06187	0.036	2.21E-05	-0.032	0.214	0.001	0.00097
TG	rs289714	rs714681	16:55564952	20:824578	0.177	0.093	0.000	0.98072	-0.020	0.11347	0.064	0.214	0.001	0.00098
TG	rs2227631	rs11570892	7:100556258	8:19867897	0.41	0.158	0.020	0.00561	-0.012	0.36775	-0.039	0.215	0.001	0.00099
TG	rs2074754	rs799168	7:72529690	7:72688800	0.481	0.221	0.004	0.59109	-0.067	2.45E-08	0.034	0.216	0.001	0.001

Column Name	Definition
Lipid	Lipid trait outcome
V1	SNP 1 in the model
V2	SNP 2 in the model
V1|chr:bp	SNP 1 chromosomal position
V2|chr:bp	SNP 2 chromosomal position
V1|MAF	SNP 1 minor allele frequency
V2|MAF	SNP 2 minor allele frequency
V1|Beta	SNP 1 beta value from the full regression model
V1|pval	SNP 1 p-value from the full regression model
V2|Beta	SNP2 beta value from the full regression model
V2|pval	SNP 2 p-value from the full regression model
Int|Beta	Beta value for SNP1-SNP2 interaction term in full regression model
Full|Rsq	R^2 value for the full regression model
Diff|Rsq	Difference in R^2 for full model and reduced regression models
lrt|pval	Likelihood ratio test p-value comparing full and reduced regression models

S2 Table: Discovery results for all models that passed replication thresholds for Biofilter analysis. Column header definitions provided at the end.

Lipid	V1	V2	V1|chr:bp	V2|chr:bp	V1|MAF	V2|MAF	V1|Beta	V1|pval	V2|Beta	V2|pval	Int|Beta	Full|Rsq	Diff|Rsq	lrt|pval
HDL	rs4148872	rs2395269	6:32910785	6:32925752	0.152	0.145	-0.023	0.00203	-0.012	0.12281	0.053	0.284	0.001	5.66E-06
HDL	rs2395269	rs4148872	6:32925752	6:32910785	0.145	0.152	-0.012	0.12281	-0.023	0.00203	0.053	0.284	0.001	5.66E-06
HDL	rs2071481	rs4148872	6:32927843	6:32910785	0.111	0.152	-0.010	0.21873	-0.020	0.00610	0.049	0.283	0.001	9.76E-05
HDL	rs4148872	rs2071481	6:32910785	6:32927843	0.152	0.111	-0.020	0.00610	-0.010	0.21873	0.049	0.283	0.001	9.76E-05
HDL	rs11757379	rs4987764	6:33650639	18:59059112	0.233	0.076	-0.023	5.85E-05	-0.032	0.00336	0.053	0.284	0.001	0.00012
HDL	rs10047459	rs595049	11:116227036	11:116204655	0.162	0.363	0.009	0.34265	0.021	0.00012	-0.033	0.284	0.001	0.00012
HDL	rs11757379	rs3826622	6:33650639	18:59054949	0.233	0.075	-0.023	6.57E-05	-0.031	0.00409	0.052	0.284	0.001	0.00014
HDL	rs11757379	rs4987752	6:33650639	18:59068813	0.233	0.076	-0.023	6.52E-05	-0.031	0.00380	0.052	0.284	0.001	0.00014
HDL	rs10047459	rs2542052	11:116227036	11:116205194	0.162	0.372	0.010	0.29667	0.018	0.00107	-0.033	0.284	0.001	0.00014
HDL	rs10047462	rs2542052	11:116227251	11:116205194	0.103	0.372	0.025	0.08481	0.016	0.00213	-0.042	0.284	0.001	0.00018
HDL	rs2860173	rs7323191	19:7080086	13:109222076	0.088	0.154	0.023	0.01107	0.003	0.68633	-0.056	0.283	0.001	0.00030
HDL	rs1549616	rs7323191	19:7083570	13:109222076	0.089	0.154	0.023	0.01153	0.003	0.69160	-0.055	0.283	0.001	0.00035
HDL	rs2860173	rs7981705	19:7080086	13:109229892	0.088	0.157	0.022	0.01398	0.006	0.38130	-0.053	0.283	0.001	0.00055
HDL	rs2393550	rs10875910	12:118582391	12:47688660	0.183	0.338	0.030	0.00030	0.015	0.00835	-0.030	0.283	0.001	0.00063
HDL	rs1549616	rs7981705	19:7083570	13:109229892	0.089	0.157	0.022	0.01443	0.006	0.38499	-0.052	0.283	0.001	0.00063
HDL	rs689243	rs595049	11:116227903	11:116204655	0.368	0.363	0.019	0.00727	0.024	0.00041	-0.023	0.283	0.001	0.00068
HDL	rs10047462	rs595049	11:116227251	11:116204655	0.103	0.363	0.019	0.19553	0.018	0.00040	-0.038	0.284	0.001	0.00071
HDL	rs10875910	rs11763144	12:47688660	7:151114829	0.338	0.139	-0.004	0.42123	-0.023	0.01113	0.033	0.283	0.001	0.00072
HDL	rs2075937	rs340807	22:35660028	3:3090514	0.442	0.230	-0.011	0.04557	-0.020	0.02036	0.025	0.283	0.001	0.00074
HDL	rs3129875	rs615672	6:32515446	6:32682149	0.290	0.358	-0.028	0.00031	-0.008	0.25701	0.026	0.283	0.001	0.00078
HDL	rs689243	rs2542052	11:116227903	11:116205194	0.368	0.372	0.019	0.00707	0.021	0.00205	-0.022	0.283	0.001	0.00087
HDL	rs11539471	rs12747412	5:118888837	1:53275870	0.076	0.398	0.040	0.00154	0.004	0.41731	-0.040	0.284	0.001	0.00088
LDL	rs10940495	rs6718902	5:55298417	2:191546449	0.286	0.244	0.035	0.03604	0.037	0.04408	-0.084	0.056	0.001	5.15E-05
LDL	rs4648038	rs11227247	4:103724227	11:65179429	0.066	0.126	-0.035	0.20300	0.020	0.30017	0.202	0.057	0.001	7.37E-05
LDL	rs4648038	rs7101916	4:103724227	11:65187936	0.066	0.125	-0.034	0.21390	0.020	0.29506	0.201	0.057	0.001	8.24E-05
LDL	rs4648038	rs2306365	4:103724227	11:65183922	0.066	0.125	-0.034	0.21488	0.021	0.28197	0.200	0.057	0.001	8.62E-05
LDL	rs6870870	rs6718902	5:55330085	2:191546449	0.422	0.244	0.036	0.01602	0.053	0.01326	-0.075	0.056	0.001	9.97E-05
LDL	rs12753665	rs4821544	1:181812684	22:35588449	0.326	0.323	0.054	0.00240	0.051	0.00418	-0.072	0.056	0.001	0.00017
LDL	rs11811630	rs4821544	1:181800533	22:35588449	0.470	0.323	0.051	0.00217	0.067	0.00138	-0.067	0.056	0.001	0.00019
LDL	rs4648038	rs3741378	4:103724227	11:65165513	0.066	0.129	-0.032	0.24985	0.020	0.29236	0.188	0.057	0.001	0.00020
LDL	rs11976696	rs12603538	7:55199827	17:70809662	0.215	0.210	-0.047	0.00829	-0.034	0.06364	0.090	0.056	0.001	0.00021
LDL	rs11976696	rs9892996	7:55199827	17:70814812	0.215	0.209	-0.046	0.00888	-0.033	0.06869	0.090	0.056	0.001	0.00024
LDL	rs4073237	rs4299376	2:43903376	2:43926080	0.065	0.312	-0.064	0.03397	0.073	5.50E-08	0.149	0.059	0.001	0.00041
LDL	rs4299376	rs4073237	2:43926080	2:43903376	0.312	0.065	0.073	5.50E-08	-0.064	0.03397	0.149	0.059	0.001	0.00041
LDL	rs4648038	rs1466462	4:103724227	11:65175940	0.066	0.374	0.114	0.00149	-0.005	0.72326	-0.123	0.056	0.001	0.00042
LDL	rs6584351	rs3747811	10:101960987	8:42248662	0.489	0.479	-0.037	0.06114	-0.052	0.00986	0.059	0.056	0.001	0.00047
LDL	rs10783298	rs11768925	12:47671224	7:151143782	0.090	0.272	-0.052	0.05620	-0.027	0.06452	0.115	0.056	0.001	0.00048
LDL	rs10783298	rs7782177	12:47671224	7:151111651	0.090	0.325	-0.058	0.04262	-0.009	0.49411	0.108	0.056	0.001	0.00053
LDL	rs1982673	rs8079544	18:58948995	17:7520777	0.155	0.054	-0.023	0.18797	-0.069	0.02270	0.168	0.056	0.001	0.00073
LDL	rs1042265	rs1005793	19:54163632	18:59041140	0.098	0.066	0.013	0.53076	-0.012	0.65099	0.197	0.056	0.001	0.00074
LDL	rs10783298	rs11773373	12:47671224	7:151156399	0.090	0.258	-0.046	0.08209	-0.016	0.28703	0.113	0.056	0.001	0.00075
LDL	rs10783298	rs6975614	12:47671224	7:151147009	0.090	0.281	-0.051	0.06202	-0.019	0.18825	0.109	0.056	0.001	0.00083
LDL	rs11089810	rs340807	22:35653584	3:3090514	0.093	0.229	-0.051	0.04805	-0.022	0.15011	0.115	0.056	0.001	0.00084
LDL	rs4073237	rs6544718	2:43903376	2:43958429	0.065	0.217	-0.066	0.01785	-0.014	0.35255	0.155	0.056	0.001	0.00091
TC	rs12753665	rs4821544	1:181812684	22:35588449	0.326	0.326	0.047	0.01384	0.055	0.00413	-0.081	0.083	0.001	8.05E-05
TC	rs11811630	rs4821544	1:181800533	22:35588449	0.468	0.326	0.051	0.00419	0.072	0.00153	-0.074	0.083	0.001	0.00012
TC	rs2075110	rs9892996	7:55186653	17:70814812	0.472	0.212	0.027	0.09433	0.077	0.00290	-0.079	0.082	0.001	0.00038
TC	rs2075110	rs12603538	7:55186653	17:70809662	0.472	0.213	0.027	0.09297	0.076	0.00315	-0.078	0.083	0.001	0.00041
TC	rs11976696	rs12603538	7:55199827	17:70809662	0.215	0.213	-0.040	0.03566	-0.036	0.06599	0.092	0.083	0.001	0.00046
TC	rs1042265	rs1005793	19:54163632	18:59041140	0.098	0.065	0.018	0.42089	-0.031	0.27827	0.221	0.083	0.001	0.00047
TC	rs10940495	rs6718902	5:55298417	2:191546449	0.288	0.245	0.035	0.04948	0.023	0.24429	-0.078	0.084	0.001	0.00050
TC	rs11976696	rs9892996	7:55199827	17:70814812	0.215	0.212	-0.040	0.03749	-0.035	0.07075	0.091	0.083	0.001	0.00052
TC	rs6584351	rs3747811	10:101960987	8:42248662	0.489	0.477	-0.027	0.20332	-0.066	0.00230	0.063	0.084	0.001	0.00053
TC	rs4648038	rs1466462	4:103724227	11:65175940	0.065	0.377	0.110	0.00437	-0.006	0.67168	-0.130	0.083	0.001	0.00053
TC	rs4648038	rs3741378	4:103724227	11:65165513	0.065	0.129	-0.042	0.15738	0.021	0.31670	0.187	0.084	0.001	0.00062
TC	rs4648038	rs2306365	4:103724227	11:65183922	0.065	0.123	-0.041	0.16517	0.024	0.24440	0.189	0.084	0.001	0.00062

S2 Table, cont’d.

Lipid	V1	V2	V1|chr:bp	V2|chr:bp	V1|MAF	V2|MAF	V1|Beta	V1|pval	V2|Beta	V2|pval	Int|Beta	Full|Rsq	Diff|Rsq	lrt|pval
TC	rs4648038	rs7101916	4:103724227	11:65187936	0.065	0.123	-0.041	0.16499	0.024	0.24208	0.189	0.084	0.001	0.00062
TC	rs4648038	rs11227247	4:103724227	11:65179429	0.065	0.124	-0.041	0.16514	0.023	0.25926	0.187	0.084	0.001	0.00069
TC	rs11591741	rs3747811	10:101966491	8:42248662	0.417	0.477	-0.028	0.19970	-0.057	0.00466	0.061	0.084	0.001	0.00084
TC	rs4148202	rs4299376	2:43921323	2:43926080	0.443	0.315	0.054	0.00192	0.132	1.43E-10	-0.069	0.085	0.001	0.00087
TC	rs4299376	rs4148202	2:43926080	2:43921323	0.315	0.443	0.132	1.43E-10	0.054	0.00192	-0.069	0.085	0.001	0.00087
TC	rs1010104	rs1005793	19:54152727	18:59041140	0.114	0.065	0.021	0.31842	-0.031	0.28551	0.199	0.083	0.001	0.00089
TC	rs11667200	rs1005793	19:54147737	18:59041140	0.113	0.065	0.026	0.21904	-0.031	0.27641	0.201	0.083	0.001	0.00090
TC	rs11597086	rs3747811	10:101943695	8:42248662	0.418	0.477	-0.028	0.20502	-0.056	0.00500	0.060	0.083	0.001	0.00097
TC	rs10783298	rs11768925	12:47671224	7:151143782	0.091	0.269	-0.064	0.02947	-0.025	0.11755	0.117	0.083	0.001	0.00098
TG	rs12099358	rs1729409	11:116231258	11:116178978	0.154	0.470	0.100	5.41E-13	0.023	0.00143	-0.074	0.216	0.002	4.51E-10
TG	rs888245	rs1729409	11:116228947	11:116178978	0.154	0.470	0.102	2.81E-13	0.022	0.00163	-0.074	0.216	0.002	7.62E-10
TG	rs11216162	rs1729409	11:116233487	11:116178978	0.182	0.470	0.082	6.74E-10	0.022	0.00276	-0.062	0.215	0.002	2.61E-08
TG	rs625145	rs1729409	11:116233146	11:116178978	0.199	0.470	0.081	6.38E-10	0.021	0.00501	-0.057	0.215	0.002	1.60E-07
TG	rs10047459	rs2542052	11:116227036	11:116205194	0.163	0.372	-0.011	0.39925	-0.010	0.17534	0.054	0.216	0.001	4.23E-06
TG	rs2542052	rs1263173	11:116205194	11:116186218	0.372	0.278	-0.020	0.02053	-0.020	0.08521	0.045	0.215	0.001	4.24E-06
TG	rs11216162	rs2542052	11:116233487	11:116205194	0.182	0.372	0.062	1.90E-07	0.031	5.38E-05	-0.051	0.215	0.001	8.49E-06
TG	rs10047459	rs595049	11:116227036	11:116204655	0.163	0.363	-0.008	0.54047	-0.012	0.11890	0.051	0.216	0.001	1.55E-05
TG	rs11216162	rs1263173	11:116233487	11:116186218	0.182	0.278	0.051	2.69E-06	0.041	1.12E-06	-0.050	0.215	0.001	3.74E-05
TG	rs12099358	rs2542052	11:116231258	11:116205194	0.154	0.372	0.067	1.67E-08	0.029	6.63E-05	-0.051	0.215	0.001	5.24E-05
TG	rs625145	rs2542052	11:116233146	11:116205194	0.199	0.372	0.060	3.55E-07	0.029	0.00023	-0.044	0.215	0.001	7.68E-05
TG	rs595049	rs1263173	11:116204655	11:116186218	0.363	0.278	-0.018	0.03980	-0.012	0.28608	0.038	0.215	0.001	9.32E-05
TG	rs888245	rs2542052	11:116228947	11:116205194	0.154	0.372	0.069	1.03E-08	0.029	8.30E-05	-0.050	0.215	0.001	9.48E-05
TG	rs11216162	rs595049	11:116233487	11:116204655	0.182	0.363	0.055	3.10E-06	0.025	0.00083	-0.043	0.215	0.001	0.00016
TG	rs10047459	rs1729409	11:116227036	11:116178978	0.163	0.470	-0.014	0.40159	-0.022	0.00262	0.046	0.215	0.001	0.00017
TG	rs6823529	rs845558	4:111068957	7:55215082	0.066	0.433	0.060	0.00192	0.013	0.05071	-0.065	0.214	0.001	0.00018
TG	rs625145	rs595049	11:116233146	11:116204655	0.199	0.363	0.056	7.24E-07	0.026	0.00062	-0.042	0.215	0.001	0.00018
TG	rs2280233	rs17881438	2:191558811	17:37792526	0.472	0.068	0.011	0.09136	0.067	0.00087	-0.063	0.214	0.001	0.00020
TG	rs4141078	rs845558	4:111060316	7:55215082	0.069	0.433	0.062	0.00100	0.013	0.05134	-0.063	0.214	0.001	0.00024
TG	rs2298986	rs845558	4:111105598	7:55215082	0.065	0.433	0.057	0.00329	0.013	0.05460	-0.064	0.214	0.001	0.00025
TG	rs2280233	rs17593222	2:191558811	17:37766516	0.472	0.069	0.011	0.09984	0.064	0.00129	-0.062	0.214	0.001	0.00027
TG	rs10047462	rs1729409	11:116227251	11:116178978	0.103	0.470	-0.003	0.89896	-0.019	0.00618	0.055	0.216	0.001	0.00027
TG	rs10047462	rs2542052	11:116227251	11:116205194	0.103	0.372	0.001	0.95130	-0.008	0.23958	0.055	0.216	0.001	0.00029
TG	rs6823529	rs13222385	4:111068957	7:55219087	0.066	0.383	0.052	0.00418	0.023	0.00042	-0.062	0.214	0.001	0.00032
TG	rs888245	rs595049	11:116228947	11:116204655	0.154	0.363	0.064	6.70E-08	0.025	0.00065	-0.045	0.215	0.001	0.00041
TG	rs2298986	rs4947978	4:111105598	7:55153950	0.065	0.359	-0.045	0.01281	0.001	0.89939	0.064	0.214	0.001	0.00046
TG	rs17133921	rs2304973	11:74712634	17:4588971	0.084	0.084	-0.014	0.23815	-0.021	0.08045	0.096	0.214	0.001	0.00047
TG	rs12099358	rs595049	11:116231258	11:116204655	0.154	0.363	0.062	2.11E-07	0.024	0.00076	-0.044	0.215	0.001	0.00047
TG	rs10745833	rs4233533	12:97580256	1:15701774	0.443	0.311	-0.021	0.01272	-0.020	0.06700	0.033	0.214	0.001	0.00047
TG	rs2280233	rs2306580	2:191558811	17:37745206	0.472	0.070	0.010	0.10857	0.060	0.00268	-0.059	0.214	0.001	0.00050
TG	rs10745833	rs4233535	12:97580256	1:15717784	0.443	0.312	-0.021	0.01334	-0.019	0.07630	0.033	0.214	0.001	0.00052
TG	rs10745833	rs4645989	12:97580256	1:15722930	0.443	0.311	-0.021	0.01390	-0.019	0.08020	0.032	0.214	0.001	0.00054
TG	rs11568993	rs2017000	4:111116764	7:55210103	0.080	0.279	0.042	0.00451	0.011	0.13283	-0.061	0.214	0.001	0.00057
TG	rs11569017	rs845558	4:111121560	7:55215082	0.060	0.433	0.053	0.00772	0.012	0.06461	-0.063	0.214	0.001	0.00059
TG	rs9991367	rs845558	4:111113750	7:55215082	0.068	0.433	0.051	0.00691	0.012	0.06345	-0.059	0.214	0.001	0.00064
TG	rs3771300	rs17881438	2:191543841	17:37792526	0.497	0.068	0.010	0.11515	0.064	0.00189	-0.058	0.214	0.001	0.00068
TG	rs2860183	rs7997595	19:7140375	13:109228769	0.371	0.160	0.025	0.00074	0.033	0.00651	-0.041	0.214	0.001	0.00068
TG	rs6823529	rs4947978	4:111068957	7:55153950	0.066	0.359	-0.041	0.02146	0.001	0.87731	0.061	0.214	0.001	0.00069
TG	rs10865222	rs3760760	2:47238686	19:51821762	0.450	0.127	0.007	0.28534	0.037	0.01198	-0.044	0.214	0.001	0.00072
TG	rs3785309	rs10742583	16:152649	11:5205217	0.096	0.160	0.025	0.04647	0.014	0.12229	-0.067	0.213	0.001	0.00073
TG	rs10208033	rs17881438	2:191587662	17:37792526	0.411	0.068	-0.016	0.01260	-0.041	0.02926	0.058	0.214	0.001	0.00078
TG	rs10208033	rs17593222	2:191587662	17:37766516	0.411	0.069	-0.016	0.01392	-0.041	0.02608	0.058	0.214	0.001	0.00081
TG	rs9991367	rs12535226	4:111113750	7:55123913	0.068	0.482	0.057	0.00579	0.011	0.08752	-0.057	0.214	0.001	0.00085
TG	rs625145	rs1263173	11:116233146	11:116186218	0.199	0.278	0.047	1.35E-05	0.037	1.94E-05	-0.039	0.215	0.001	0.00085
TG	rs2860184	rs9521510	19:7238748	13:109224872	0.367	0.330	-0.020	0.02166	-0.031	0.00103	0.031	0.214	0.001	0.00094
TG	rs6823529	rs9692301	4:111068957	7:55211248	0.066	0.303	0.043	0.01055	0.014	0.04273	-0.062	0.213	0.001	0.00096
TG	rs2298986	rs12535226	4:111105598	7:55123913	0.065	0.482	0.058	0.00562	0.011	0.09343	-0.058	0.214	0.001	0.00099
TG	rs2237054	rs845558	4:111130638	7:55215082	0.068	0.433	0.052	0.00600	0.012	0.07114	-0.057	0.214	0.001	0.00099
TG	rs11231741	rs4645887	11:63803461	19:54151688	0.316	0.376	0.018	0.06858	0.008	0.34234	-0.031	0.214	0.001	0.00099

Column Name	Definition
Lipid	Lipid trait outcome
V1	SNP 1 in the model
V2	SNP 2 in the model
V1|chr:bp	SNP 1 chromosomal position
V2|chr:bp	SNP 2 chromosomal position
V1|MAF	SNP 1 minor allele frequency
V2|MAF	SNP 2 minor allele frequency
V1|Beta	SNP 1 beta value from the full regression model
V1|pval	SNP 1 p-value from the full regression model
V2|Beta	SNP2 beta value from the full regression model
V2|pval	SNP 2 p-value from the full regression model
Int|Beta	Beta value for SNP1-SNP2 interaction term in full regression model
Full|Rsq	R^2 value for the full regression model
Diff|Rsq	Difference in R^2 for full model and reduced regression models
lrt|pval	Likelihood ratio test p-value comparing full and reduced regression models
